# Chromosomal rearrangements and protein globularity changes in *Mycobacterium tuberculosis* isolates from cerebrospinal fluid

**DOI:** 10.7717/peerj.2484

**Published:** 2016-09-21

**Authors:** Seow Hoon Saw, Joon Liang Tan, Xin Yue Chan, Kok Gan Chan, Yun Fong Ngeow

**Affiliations:** 1 Department of Pre-Clinical Sciences, Faculty of Medicine and Health Sciences, Universiti Tunku Abdul Rahman, Bandar Sungai Long, Malaysia; 2 Department of Biomedical Science, Faculty of Science, Universiti Tunku Abdul Rahman, Kampar, Perak, Malaysia; 3 Department of Medical Microbiology, Faculty of Medicine, University of Malaya, Kuala Lumpur, Malaysia; 4 Faculty of Information Science and Technology, Multimedia University, Melaka, Malaysia; 5 Division of Genetics and Molecular Biology, Institute of Biological Sciences, Faculty of Science, University of Malaya, Kuala Lumpur, Malaysia

**Keywords:** Meningitis, Comparative genomics, *Mycobacterium tuberculosis*

## Abstract

**Background:**

Meningitis is a major cause of mortality in tuberculosis (TB). It is not clear what factors promote central nervous system invasion and pathology but it has been reported that certain strains of *Mycobacterium tuberculosis* (*Mtb*) might have genetic traits associated with neurotropism.

**Methods:**

In this study, we generated whole genome sequences of eight clinical strains of *Mtb* that were isolated from the cerebrospinal fluid (CSF) of patients presenting with tuberculous meningitis (TBM) in Malaysia, and compared them to the genomes of H37Rv and other respiratory *Mtb* genomes either downloaded from public databases or extracted from local sputum isolates. We aimed to find genomic features that might be distinctly different between CSF-derived and respiratory *Mtb*.

**Results:**

Genome-wide comparisons revealed rearrangements (translocations, inversions, insertions and deletions) and non-synonymous SNPs in our CSF-derived strains that were not observed in the respiratory *Mtb* genomes used for comparison. These rearranged segments were rich in genes for PE (proline-glutamate)/PPE (proline-proline-glutamate), transcriptional and membrane proteins. Similarly, most of the ns SNPs common in CSF strains were noted in genes encoding PE/PPE proteins. Protein globularity differences were observed among mycobacteria from CSF and respiratory sources and in proteins previously reported to be associated with TB meningitis. Transcription factors and other transcription regulators featured prominently in these proteins. Homologs of proteins associated with *Streptococcus pneumoniae* meningitis and *Neisseria meningitidis* virulence were identified in neuropathogenic as well as respiratory mycobacterial spp. examined in this study.

**Discussion:**

The occurrence of in silico genetic differences in CSF-derived but not respiratory *Mtb* suggests their possible involvement in the pathogenesis of TBM. However, overall findings in this comparative analysis support the postulation that TB meningeal infection is more likely to be related to the expression of multiple virulence factors on interaction with host defences than to CNS tropism associated with specific genetic traits.

## Background

Tuberculosis (TB) is an ancient communicable disease that has persisted throughout the ages to remain a major killer of the human race. The main portal of entry for the causative pathogen, *Mycobacterium tuberculosis* (*Mtb*) is the respiratory tract. Following inhalation into the pulmonary alveoli, these bacilli are phagocytosed by alveolar macrophages in which they survive to either cause local lesions or be disseminated to extra-pulmonary sites (Smith, 2003).

Tuberculous meningitis (TBM) is a severe form of extrapulmonary TB that is associated with high morbidity and mortality (Erhabor, Adewole & Ogunlade, 2006; Bidstrup et al., 2002). In parts of the world where the incidence of TB is high, TBM may occur in more than 10% of TB cases, especially among children and HIV infected individuals (Ige, Sogaolu & Ogunlade, 2005). The pathogenesis of this central nervous system (CNS) *Mtb* infection is still not clear. Both host susceptibility factors and specific mycobacterial genetic traits have been implicated. The former has been documented extensively by clinical reports on the greater risk of CNS infection in immunocompromised hosts (Keane et al., 2001; Vinnard & Macgregor, 2009; Elmas, Akinci & Bilir, 2011). Furthermore, various polymorphisms in human genes have been identified to be more strongly associated with susceptibility to meningeal than pulmonary TB (Hawn et al., 2006; Campo et al., 2015; Hoal-Van Helden et al., 1999). On the other hand, in vitro and animal model studies have provided evidence that some *Mtb* strains are better able to invade the CNS because of greater production of neurotropic factors (Huang & Jong, 2001). *Mtb* strains causing meningitis have been associated with distinct genotypes (Arvanitakis et al., 1998; de Viedma et al., 2006, Caws et al., 2008; Hesseling et al., 2010). At the Indian National Institute of Mental Health and Neurosciences, several unique DNA patterns were shown to be present in cerebrospinal fluid (CSF) *Mtb* isolates that were not present in the *Mtb* DNA pattern library made up of 23,000 strains from across the world (NIMHANS, 2010). Pando et al. (2010) reported that BALB/c mice infected via the intra-tracheal route by *Mtb* came down with meningitis when infected with isolates from CSF but not with isolates from sputum. Likewise, Be et al. (2008) identified five *Mtb* genes (Rv0311, Rv0805, Rv0931c, Rv0986, and MT3280) associated with invasion or survival in the CNS but not in lung tissues. In particular, the sensor domain of *Mtb* pknD (Rv0931c) was able to trigger the invasion of brain endothelia but not the lung epithelia (Be, Bishai & Jain, 2012).

The collective evidence from patient and microbial studies indicate that both host and microbial factors direct the pathogenesis of TBM. The importance of host-microbe interactions in this infection is illustrated in a number of studies. Caws et al. (2006) showed an association between the Beijing genotype and HIV status in Vietnamese patients with TBM and linked individuals with a TLR2 T597C mutation to a greater likelihood of infection with the East-Asian/Beijing genotype. Similarly, Reed et al. (2004) suggested that the virulence of the Beijing *Mtb* genotype is due to the presence of an intact polyketide synthase gene (*pks 15/1*) encoding a phenolic glycolipid (PGL) that is able to inhibit the release of the pro-inflammatory cytokines, tumour-necrosis factor-alpha and interleukins 6 and 12. It has been observed that *Mtb* of Euro-American lineage that do not have an intact *pks15/1* gene are less capable of causing extrapulmonary TB including TBM (Caws et al., 2006). Hence, while host genotype determines susceptibility to infection by different *Mtb* strains, microbial virulence factors can influence disease manifestation by their specific interactions with host immune response.

In this study, we took advantage of whole genome sequencing technology and the accessibility to public *Mtb* genome databases to compare genomic features between *Mtb* isolates from CSF and respiratory specimens. We hoped to find polymorphisms that might extend existing knowledge on CNS tropism in clinical TB.

## Materials and Methods

This study was approved by the Medical Ethics Committee, University Malaya Medical Centre, Kuala Lumpur, Malaysia (Reference no. 975.28).

### Bacterial strains

The *Mtb* strains, UM-CSF01, UM-CSF04, UM-CSF05, UM-CSF06, UM-CSF08, UM-CSF09, UM-CSF15 and UM-CSF17 (hereafter referred to collectively as UM-CSF strains) were CSF isolates from patients treated for TBM in the University Malaya Medical Center, Kuala Lumpur, Malaysia. They were recovered from routine cultures in the BACTEC MGIT 960 liquid culture system (Becton Dickinson) and identified using a reverse line probe hybridisation assay (GenoType *Mycobacterium* CM/AS; Hain Lifescience GmbH, Germany). The corresponding sputum samples for these strains were culture negative for *Mtb*. The genotypes identified using the spoligotyping kit from Ocimum Biosolutions (Hyderabad, India) were Beijing ST1 (UM-CSF01, UM-CSF05, UM-CSF08, UM-CSF09 and UM-CSF17), EAI_IND (UM-CSF06), Unknown (UM-CSF04) and H3 ST50 (UM-CSF15). They were kept in Middlebrook 7H9 broth with 15% glycerol, at −80 °C, until required for further testing. For whole-genome sequencing, they were subcultured on Lowenstein-Jensen slants, heat inactivated at 80 °C for 2 h, and cooled down to room temperature before they were used for DNA extraction.

### Extraction of genomic DNA

Bacterial genomic DNA was extracted with the Phenol/Chloroform/Isoamylalcohol (PCI) method (Sambrook, Fritsch & Maniatis, 1989) to obtain a high yield of DNA. Briefly, the inactivated *Mtb* culture was centrifuged to a maximum speed for 15 min. The pellet obtained was lysed by incubation in 10 mg/ml of lysozyme overnight at 37 °C, followed by the addition of 10% SDS and inactivation of DNases and RNases with 10 mg/ml Proteinase K. The mixture was vortex mixed and incubated at 55 °C for 5 h. Before the purification of nucleic acid, 5 M of sodium chloride was added, followed by the addition of phenol/chloroform/isoamyl alcohol (25:24:1) to remove all proteins. The mixture was then centrifuged at maximum speed. The aqueous supernatant containing DNA was transferred to a microcentrifuge tube and the purification step was repeated. Finally, nucleic acids were recovered from aqueous solution with ethanol precipitation using 3 M of sodium acetate and ice-cold isopropanol, and overnight incubation of the mixture at −20 °C. The pellet was washed with 80% ethanol and dried at room temperature. The required DNA precipitate was dissolved and diluted with autoclaved distilled water and its concentration and purity were measured in a spectrophotometer at OD_260_ (Nanophotometer, Implen USA).

### Whole genome sequencing

#### Library preparation and sequencing

The DNA sequencing libraries were prepared using Nextera− DNA Sample Preparation kit (Illumina, San Diego, CA, USA). The quality of DNA library was validated by Bioanalyzer 2,100 using high sensitivity DNA kit (Agilent, USA) prior to sequencing. Upon sequencing, DNA (6 pM) was loaded into the sequencing cartridge and the sequencing was performed on the Illumina MiSeq platform.

#### Read quality assessment, assembly and annotation

The quality of raw sequences generated from MiSeq was checked using FastQC. Raw reads were trimmed at Phred probability score of 30 and were de novo assembled using CLC Genomic Workbench 5.1 (Qiagen Inc., Venlo, The Netherlands). Trimmed sequences were assembled with length fraction of 0.8 and similarity fraction of 0.8. All assemblies were evaluated based on statistical assessment, focusing on genome size, sequence continuity and number of contigs. The genomes were further screened for contamination against common contaminants databases and then used for downstream analyses.

To decrease the possibility of inaccurate assembly, we repeated the assembly and scaffolding of the genomes in IDBA-UD (Peng et al., 2012) and SSPACE (Boetzer et al., 2011) respectively.

The Whole Genome Shotgun project for UM-CSF01 and UM-CSF05 has been deposited at DDBJ/ENA/GenBank under the accessions LLXF00000000, LLXG00000000, respectively and that for UM-CSF04, UM-CSF06, UM-CSF08, UM-CSF09, UM-CSF15 and UM-CSF17 under the accessions LXGA00000000, LXGB00000000, LXGC00000000, LXGD00000000, LXGE00000000 and LXGF00000000, respectively.

### Genomic analysis

To reduce the likelihood of observing traits peculiar to local strains, we included respiratory strains from Malaysian patients for comparison. Hence, in addition to the UM-CSF strains, we included for analysis, 13 other *Mtb* sputum isolates of patients managed in our medical centre over the same time period (2009–2011). All 13 strains were de novo sequenced (using Illumina Miseq technology) and annotated for another project currently in progress in our laboratory. For their use in this study, we reconstructed their genomes in IDBA-UD 1.0.9.

#### Genome rearrangements

We used Gepard (Krumsiek, Arnold & Rattei, 2007) and Mauve (Darling et al., 2004) to gain an overview of chromosomal structural variation between the UM-CSF strains, the H37Rv reference strain, the 16 genomes (Table S1) downloaded on 6th August 2015, from Genome Database (one representative from each group as defined in the database) (http://www.ncbi.nlm.nih.gov/genome/genomegroups/166?) and the 13 local sputum strains.

This was followed by pairwise-alignment and comparisons using Mugsy (Angiuoli & Salzberg, 2011), with default parameters, to identify the positions of rearranged regions in UM-CSF strains.

#### Identification of polymorphisms

For this analysis, we downloaded from NCBI’s SRA database, the sequencing reads for 56 *Mtb* genomes reported to be from sputum strains (Table S2). These reads and those from our UM-CSF and respiratory strains were mapped to H37Rv using Burrow-Wheeler Aligner. Gene variants were extracted using mpileup of samtools (Li et al., 2009) and annotated with snpEff (Cingolani et al., 2012). The variants identified were filtered based on the following criteria: minimum number of good quality read of three (DP4 ≥ 3); minimum mapping quality of 25 (MQ ≥ 25); SNP and Indel quality of 20 and 60 respectively.

#### Amino acid comparisons

The assembled genomes of UM-CSF strains were annotated using the self-training annotation algorithm in GeneMarkS (Besemer, Lomsadze & Borodovsky, 2001). Orthologous protein sequences were identified in the ProteinOrtho program, with e-value of 1 × 10^−5^ (Lechner et al., 2011). The effect of amino acid substitution was evaluated using the I-mutant webserver (Capriotti, Fariselli & Casadio, 2005) for change in protein stability and the GlobPlot standalone python script (Linding et al., 2003) for globularity.

## Results

### Genome overview

The genomes of UM-CSF strains were recovered from approximately 55–92X sequencing coverage respectively. The detailed statistical measurements of the genomes are shown in Table 1. Compared to H37Rv and the other 29 respiratory *Mtb* genomes (16 downloaded from NCBI and 13 extracted from Malaysian isolates), the UM-CSF strains appeared to have fewer gene duplications but a larger number of PE (proline-glutamate)/PPE(proline-proline-glutamate)/PGRS (polymorphic GC-rich sequence) proteins. There is no notable difference between the two groups in the structure of 16S rRNA and tmRNAs and in the number of tRNAs (20–30 for CSF strains and 19–33 for respiratory strains).

**Table 1:** Statistical measurements of UM-CSF genomes.

Strain	N_50_	No. contigs	No. scaffolds	Reads used (%)	Scaffolded genome size (bp)	No. protein CDS
UM-CSF01	36,496	315	234	96.67	4,282,569	4,271
UM-CSF04	98,670	140	136	93.09	4,392,768	4,323
UM-CSF05	74,553	182	138	97.56	4,338,921	4,190
UM-CSF06	83,364	135	127	87.01	4,371,105	4,310
UM-CSF08	91,928	174	156	93.77	4,352,163	4,353
UM-CSF09	122,404	133	103	89.71	4,355,856	4,291
UM-CSF15	91,928	129	126	94.05	4,374,121	4,313
UM-CSF17	122,408	131	111	90.99	4,356,783	4,308

### Genome rearrangements

The UM-CSF strains showed structural differences from H37Rv and the 29 respiratory *Mtb* genomes used for comparison. Rearrangement analysis by Gepard and Mauve gave identical results (Figs. S1–S6). Both analyses indicated sequence fragments that could have undergone rearrangement events in six of the eight UM-CSF strains (UM-CSF01, UM-CSF05, UM-CSF06, UM-CSF09, UM-CSF15 and UM-CSF17). The affected regions ranged from 223 to 500, 211 bp in one to eight contigs and involved one to 492 genes. Based on the annotation of H37Rv, many of the genes affected belonged to PE/PPE/PGRS gene families that are known to be associated with virulence in *Mtb*. Also affected were genes encoding mammalian cell entry (*mce*) proteins, transcriptional factors, metabolic enzymes and toxin-antitoxin (TA) proteins (Tables S3–S5).

In UM-CSF01 and UM-CSF05, the recombination sites for translocations and inversions were not within genes and hence, did not affect adjacent gene sequences. In the remainder four affected UM-CSF strains, the rearrangements caused deletions resulting in gene truncations. The affected genes are Rv3425 encoding PPE57 in UM-CSF06; Rv1141c encoding enoyl-CoA hydratase EchA11 in UM-CSF09; Rv1587c coding for a hypothetical protein in UM-CSF15, and Rv 3513c encoding a fatty-acid--CoA ligase FadD18 in UM-CSF17. The function of PPE57 is unknown but both EchA11 and FadD18 are suspected to be involved in lipid metabolism, the former in fatty acid oxidation and the latter in lipid degradation.

### Micro-variants and protein globularity changes

Against the H37Rv genome, we identified 737–2,578 micro-variants from the UM-CSF strains. However, as we were interested in only micro-variants possibly associated with cerebrospinal invasion, we compared our UM-CSF strains with 69 other respiratory *Mtb* genomes (56 downloaded from the NCBI SRA database (Table S2) and 13 local respiratory *Mtb* genomes). We found 63–534 micro-variants specific to our eight UM-CSF strains within protein coding regions. None were in any of the regions of difference (RD1–RD16) where many *Mtb* strain-specific features and virulence factors are normally found. No variant was shared by all eight strains but 36 variants involving 10 genes (PE-PGRS10, PPE58, PE_PGRS49, lppD, PE_PGRS21, Rv0278c, embR, PE_PGRS19, PPE53 and PPE24) were found in at least four of the strains. The variants in eight of these genes led to amino acid changes but only two altered genes have known functions: PE_PGRS19, a putative outer membrane protein (Song et al., 2008) and embR which is involved in transcription, the biosynthesis of mycobacterial cell wall arabinan and resistance to ethambutol (Table S6).

**Figure 1:**
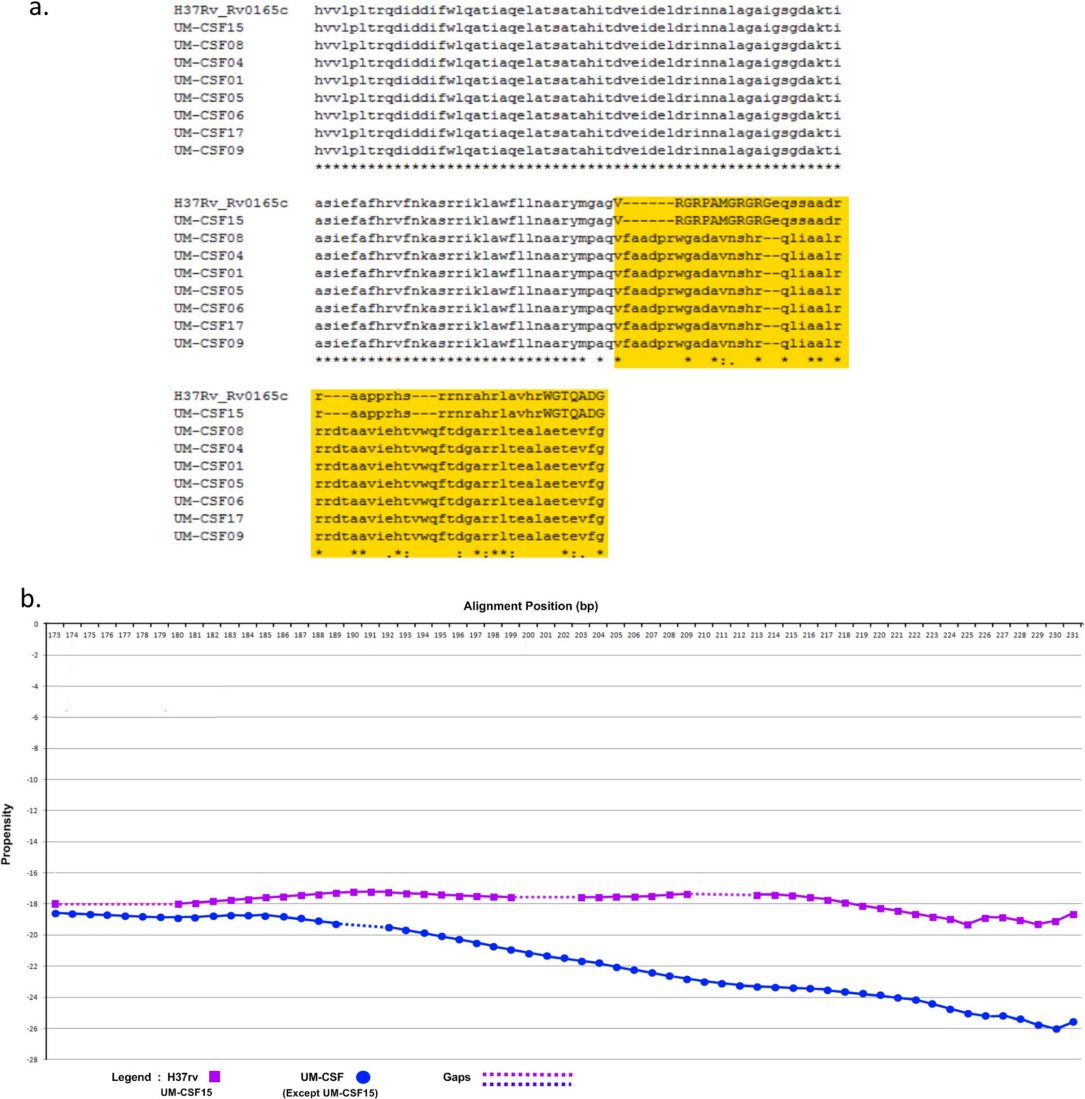
(A) Alignment of Rv0165c sequences in eight UM-CSF strains and H37Rv, showing amino acid and globularity changes. (B) Differences in propensity scores for amino acids in UM-CSF strains and H37Rv.

The amino acid sequence of a protein determines its folding into three-dimensional structures which may be ordered (globular with high hydrophobicity) or disordered (unstructured, typically with low hydrophobicity and a high proportion of polar and charged amino acids). There are many methods for predicting disordered proteins and various factors such as net charge, hydrophobicity and protein size can affect the accuracy of predictions. In UM-CSF strains, we noted 1,556 amino acid substitutions in 1,084 orthologs of proteins in H37Rv. With GlobPlot, 646 of the substitutions were predicted to be in disordered segments and 910 in globular segments. Figure 1 shows an example of the amino acid and globularity differences between UM-CSF strains and H37Rv. All proteins showing different globularity between UM-CSF strains and H37Rv were subjected to enrichment analysis using DAVID (Dennis et al., 2003) (Table S7). The single largest group of the enriched proteins involved transcription factors and transcription regulators. Four of the enriched proteins were found in three different CSF strains each. These were Rv0144 (probable transcriptional regulatory protein with Leu to Asp substitution), Rv3730c (hypothetical protein with Thr to Asp substitution), Rv0802c (possible succinyltransferase with Ser to Pro substitution) and Rv2034 (transcriptional regulatory protein with Ala to Thr substitution). All four showed higher propensity to disorder, and all, with the exception of Rv0802c, were accompanied by a decrease in stability. These changes are consistent with previous observations that amino acid substitutions leading to the acquisition of new protein functions are often accompanied by a loss of thermodynamic stability which may be compensated subsequently by the stabilizing effect of other unrelated mutations (Shoichet et al., 1995; Wang, Minasov & Shoichet, 2002; Tokuriki et al., 2008; Studer, Dessailly & Orengo, 2013). Furthermore, all four proteins were enriched (49–53%) in the disorder-promoting amino acids (A, R, G, Q, S, P, E, K) and depleted (28–34%) of order-promoting amino acids (W, C, F, I, Y, V, L, N) as described by Dunker et al. (2001). Hydrophobic amino acids form less than 50% of the amino acid sequence in each of these disordered proteins. As many disordered proteins or protein domains are functionally related to disease, it is possible that some of those identified in UM-CSF strains might contribute to an enhanced ability to cause TBM. However, a larger search for these protein structural and globularity changes found them to be present in many of our local respiratory strains as well.

### Proteins reported to be associated with meningitis in *M. tuberculosis*

We looked for putative meningitis-associated factors that had been previously reported in scientific literature. Of 63 proteins reported for *Mtb* (Av-Gay & Everett, 2000; Pethe et al., 2001; Tsenova et al., 2005; Jain et al., 2006; Be et al., 2008; Be, Bishai & Jain, 2012) (Table S8), homologs of 56–60 were found in UM-CSF strains but only two (Rv0311 encoding a hypothetical protein and Rv0619 encoding a probable galactose-1-phosphate uridylyltransferase GalTb) were found in all eight CSF strains. Compared to H37Rv, both proteins showed amino acid and globularity differences. The Rv0311 protein in five of the eight UM-CSF strains had Glu (GAG) instead of Asp (GAT) at position 119 and was predicted to be disordered. Rv0619 in all eight UM-CSF strains had a substitution of Ala (GCC) for Thr (ACC) at position 174 and appeared as a globular segment with a change from polarity to hydrophobicity.

Additionally, we performed a similar search in five other mycobacteria that are associated with neuropathology. These comprised *M. leprae* and *M. lepromatosis* that cause different forms of leprosy*, M. bovis* that is usually linked with extrapulmonary TB, and two rapid-growers *M. llatzerense* and *M. immunogenum* that had been isolated from a case of brain abscess (Greninger et al., 2015). Fifty-six homologs of the 63 meningitis-related genes from *Mtb* were identified in *M. bovis*, followed by 16 in *M. leprae,* 15 in *M. lepromatosis,* 14 in *M. llatzerense,* and 11 in *M. immunogenum.* Our UM-CSF strains shared four of the 63 meningitis-related genes (Rv0014c, Rv1837c, Rv2176 and Rv0984) with all five of these mycobacterial species and five other genes (Rv1273c, Rv2318, Rv0983, Rv0966c and Rv0805) with the three slow-growing mycobacteria (Table 2). The Rv2947c (*pks15/1*) gene was found in the UM-CSF strains, *M. leprae* and *M. ilatzerense*. In the Beijing genotype of *Mtb,* an intact *pks 15/1* is believed to be responsible for virulence and extrapulmonary disease (Reed et al., 2004). Consistent with their extrapulmonary (CNS) location in the host, five of our eight UM-CSF strains were genotyped as Beijing ST1 and each carried an intact *pks 15/1* gene.

**Table 2:** Genes shared by UM-CSF strains, other mycobacteria associated with neuropathology, *S. pneumoniae* and *N. meningitidis*.

Host	Rv number	Gene name	Product	Function
UM-CSF strains *Mtb*	Rv0311	Rv0311	Unknown protein	Unknown
UM-CSF strains *Mtb*	Rv0619	galTB	Probable galactose-1-phosphate uridylyltransferase GalTb	Galactose metabolism (leloir pathway) catalytic activity
UM-CSF strains Mycobacteria, *S pneumoniae*	Rv1699	pyrG	CTP synthase PyrG	Pyrimidine biosynthesis catalytic activity
UM-CSF strains Mycobacteria, *S pneumoniae*	Rv2606c	snzP	Pyridoxine biosynthesis protein SnzP	Biosynthesis of pyridoxine/pyridoxal 5-phosphate
UM-CSF strains Mycobacteria, *S pneumoniae*	Rv0357c	purA	Probable adenylosuccinate synthetase purA	AMP biosynthesis
UM-CSF strains Mycobacteria *N meningitidis*	Rv2457c	clpX	ATP-dependent CLP protease ATP-binding subunit ClpX	Directs the CLP protease to specific substrates; performs chaperone functions in the absence of CLP protease
UM-CSF strains *N meningitidis*	Rv2397c	cysA1	Probable sulfate-transport ATP-binding protein ABC transporter cysA1	Active transport of multiple sulfur-containing compounds across the cell membrane; energy coupling to the transport system
UM-CSF strains Mycobacteria	Rv1837c	glcB	Malate synthase G GlcB	glyoxylate bypass; alternative to TCA cycle
UM-CSF strains Mycobacteria	Rv0014c	pknB	Transmembrane serine/threonine-protein kinase B PknB	Signal transduction (via phosphorylation)
UM-CSF strains Mycobacteria	Rv2176	pknL	Probable transmembrane serine/threonine-protein kinase L PknL	Signal transduction (via phosphorylation)
UM-CSF strains Mycobacteria	Rv0984	moaB2	Possible pterin-4-alpha-carbinolamine dehydratase MoaB2	Molybdopterin biosynthesis
UM-CSF strains SG Mycobacteria	Rv1273c	Rv1273c	Probable drugs-transport transmembrane ATP-binding protein ABC transporter	Active transport of drugs across the membrane (export)
UM-CSF strains SG Mycobacteria	Rv2318*	uspC	Probable periplasmic sugar-binding lipoprotein UspC	Active transport of sugar across the membrane (import)
UM-CSF strains SG Mycobacteria	Rv0983	pepD	Probable serine protease PepD (serine proteinase)	Unknown; possibly hydrolyzes peptides and/or proteins
UM-CSF strains SG Mycobacteria	Rv0966**	Rv0966	Conserved protein	Unknown
UM-CSF strains SG Mycobacteria	Rv0805	Rv0805	Class III cyclic nucleotide phosphodiesterase	Hydrolyzes cyclic nucleotide monophosphate to nucleotide monophosphate

**Notes:**

*Mtb*, *Mycobacterium tuberculosis.*

Mycobacteria, *M. bovis, M. leprae, M. lepromatosis, M. ilatzerense, M. immunogenum.*

SG Mycobacteria, *M. bovis, M. leprae, M. lepromatosis.*

* Rv2318 is not found in CSF08.

** Rv0966 is not found in CSF04 and CSF06.

### Meningitis-associated genes from other bacterial pathogens

**Figure 2:**
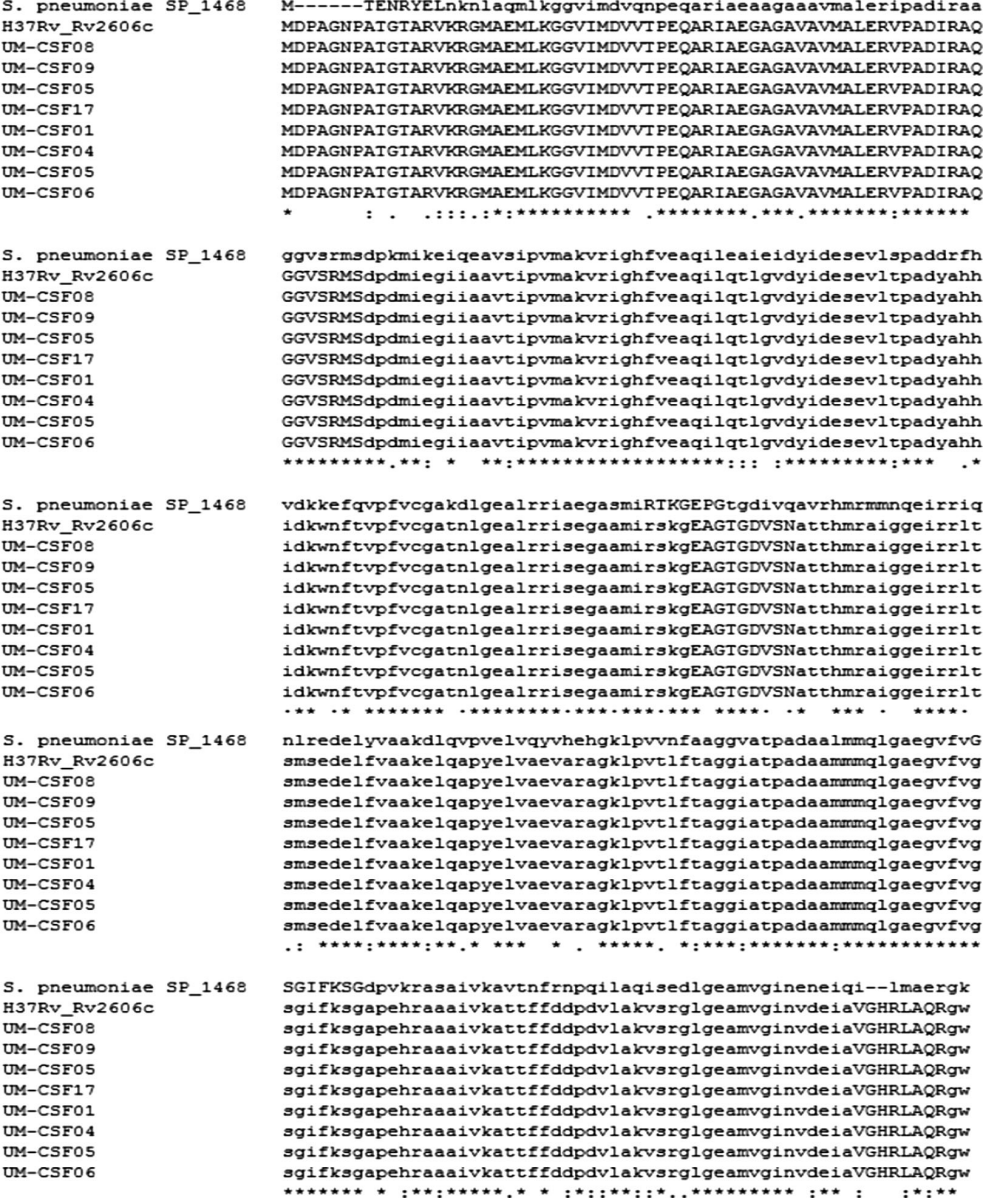
Alignment of meningitis-associated proteins from *S. pneumoniae*, H37Rv and eight UM-CSF strains.

*Streptococcus pneumoniae*, *Escherichia coli* K-1 and *Neisseria meningitidis* are pathogens known to cause meningitis in humans. Of 141 proteins reported to be associated with *S. pneumoniae* meningitis (Orihuela et al., 2004; Molzen et al., 2011; Mahdi et al., 2012) (Table S9), three, Rv1699 (CTP synthase PyrG), Rv2606c (pyridoxine biosynthesis protein SnzP) and Rv0357c (adenylosuccinate synthetase PurA) were found in our UM-CSF strains. These genes showed 51–68% sequence similarity with their homologs in *S. pneumoniae* but were identical in all UM-CSF strains and H37Rv, in protein sequence as well as globularity (Fig. 2). When compared against 164 *N. meningitidis* virulence genes reported by Hao et al. (2011) (Table S10), UM-CSF strains shared two virulence homologs with this neuropathogen: Rv2457c, encoding ATP-dependent CLP protease ATP-binding subunit clpX and Rv2397c, encoding sulfate-transport ATP-binding protein ABC transporter CysA1. We failed to identify the genes for cell surface outer membrane Opa and Opc proteins that were previously reported to confer tissue tropism in *N. meningitidis* (Virji et al., 1993) and we did not find any homologs of previously reported *E. coli* K1 neurotropic genes such as IbeA, IbeB, AslA, YijP, and OmpA (Pouttu et al., 1999; Huang et al., 2001; Yao, Xie & Kim, 2006) (Table S11), in UM-CSF strains.

## Discussion

With the aid of whole genome sequencing, comparative genomics has become the mainstay analytical platform for the study of bacterial taxonomy, evolution and virulence. Even with impaired host immunity being recognized to be mainly responsible for extra-pulmonary dissemination in TB, it is still tempting to use the vast volume of bacterial sequence data amassed to look for mycobacterial factors that might direct the dissemination of *Mtb* to the CNS. Towards this end, we compared genomic features in CSF and respiratory *Mtb* isolates and searched the genomes for genes previously reported to be associated with CNS infection.

Although *Mtb* exhibits less genetic diversity than other bacteria, comparative genomic analyses have revealed SNPs, large sequence polymorphisms and variations in mobile elements among *Mtb* isolates from different sources (Fleischmann et al., 2002; Tsolaki et al., 2004). Large-scale chromosomal rearrangements have also been reported, such as the large inversions detected in the KwaZulu-Natal (KZN) strains from South Africa (Okumura et al., 2015) and the B0/W148 strains from Russia (Shitikov et al., 2014). In our UM-CSF strains, we found translocations, inversions, indels and SNPs not detected in 30–69 respiratory strains used for comparison. Many of the genes involved were putative virulence factors that included abundant PE and PPE proteins. The prominence of PE/PPE proteins here is not surprising as these hypervariable cell surface antigens form about 10% of the total coding sequences in the *Mtb* genome (Cole et al., 1998) and their association with large sequence polymorphisms (Talarico et al., 2007) has been reported. It is believed that these uniquely mycobacterial proteins are secreted by the ESX apparatus in the cell membrane (Abdallah et al., 2006) and are involved in many aspects of the infection process, such as promoting *Mtb* entry into macrophages and evasion of host immune responses, resulting in *Mtb* dissemination and pathology in different organs and tissues. Unfortunately, all the PE/PPE proteins we identified have no known function except for PE-PGRS30 which has been listed as a virulence factor. Also abundant in the rearranged fragments are TA and mce proteins. The TA systems in *Mtb* are up-regulated following bacterial entry into macrophages (Ramage, Connolly & Cox, 2009). The VapBC family which made up most of the TA proteins in our UM-CSF strains, was shown to control the persistence of uropathogenic *E. coli* within host tissues in cases of sepsis, meningitis and urinary tract infections (Norton & Mulvey, 2012). It is possible that these proteins also contribute to the persistence of *Mtb* in TBM. Similarly, mce proteins may enhance cellular invasion and persistence of *Mtb* in the CNS by facilitating the uptake and utilisation of cholesterol from host cells during infection (Pieters & Gatfield, 2002). We looked for the 11 putative serine-threonine protein kinases (pknA-pknL) in *Mtb* as Be, Bishai & Jain (2012) identified *Mtb* pknD (Rv0931c) as a key microbial factor required for CNS tropism. We found the pkn genes in our UM-CSF strains but only pknG (Rv0410c) was in a rearranged fragment in UM-CSF05.

We looked for globularity changes as the folding of proteins affects their function. In our DAVID analysis of proteins showing different globularity between UM-CSF strains and H37Rv, transcription factors and other transcription regulators predominate. It is expected that changes in transcription-related proteins would have profound effects on gene expression resulting in wide-ranging changes in *Mtb* behavior including altered tropism and virulence. An interesting finding is the enrichment of proteins involved in androgen and estrogen metabolism. This could be related to the role of sex hormones in the modulation of bacterial-host interactions. It is known that, while sex hormones from the host can affect the metabolism and virulence of bacteria, they are also degraded by bacteria to be used as carbon and energy sources (García-Gómez, González-Pedrajo & Camacho-Arroyo, 2013; Neyrolles & Quintana-Murci, 2009). It is plausible that a change in protein domain globularity in the *Mtb* proteins involved in sex hormone metabolism could upset the usual bacterial-host interactions to facilitate CNS invasion in TBM. Among other enriched proteins of interest are ABC transporters, glycerophospholipids and proteins involved in DNA repair. ABC transporters transfer molecules across cell membranes and are implicated in *Mtb* traversal across the blood-brain barrier (Jain et al., 2006). Glycerophospholipids are found in neural membranes (Farooqui, Horrocks & Farooqui, 2000). A functional change in these lipids could increase membrane permeability to *Mtb.* Bacterial DNA can be damaged by reactive oxygen species and reactive nitrogen intermediates generated from cellular metabolism and the host immune response (Davidsen et al., 2007). DNA repair mechanisms include nucleotide excision repair (NER) controlled by the UvrABCD endonuclease enzyme complex, base excision repair and DNA mismatch repair. Darwin & Nathan (2005) showed that the *uvrB* gene is required for *Mtb* to resist reactive oxygen and nitrogen molecules in vivo. In UM-CSF strains, we found the *uvrC* gene in a rearranged fragment in UM-CSF01 and identified homologs of mismatch repair genes among the enriched proteins with globularity changes.

In our search for common neurotropic genes, we compared our UM-CSF strains with five mycobacterial species associated with neuropathology (*M. leprae, M. lepromatosis, M. bovis, M. ilatzerense* and *M. immunogenum*) and three other established neuropathogens, *S. pneumoniae, N. meningitidis* and *E. coli* K1. We didn’t find any evidence of horizontal gene transfer of previously reported neurotropic factors, between UM-CSF strains and the other eight pathogens. We did find, however, homologs of genes associated with CNS disease in all the pathogens we examined, with the exception of *E. coli* K1 (Table 2). The number of *Mtb* gene homologs was, expectedly, highest in *M. bovis,* a member of the *Mtb* complex (56 of 63, 89%), followed by *M. leprae* (16 of 63, 25%) and *M. lepromatosis* (15 of 63, 24%). To our surprise, the number of homologs in *M. llatzerense* (14 of 63, 22%) and *M. immunogenum* (11 of 63, 17%), the two environmental rapid growers isolated from a brain abscess, was almost as high as in the established neuropathogens *M. leprae* and *M. lepromatosis*.

The nine common genes we identified in UM-CSF strains and other mycobacterial spp. are mostly involved in cell membrane transport, signal transduction, nucleotide biosynthesis, and energy metabolism (Table 2). Although these functions are common to all microbial cells, most of the genes have been previously associated with TB CNS pathology. For instance, Rv2318, Rv0983, Rv0984, and Rv0966c were found to be up-regulated in an in vitro model of TB infection of the human brain microvascular endothelium (Jain et al., 2006); Rv1837c was reported to be expressed in the early stages of TBM in children (Haldar et al., 2012); the transmembrane serine/threonine-protein kinase PknD (Rv0931c) was found by Be, Bishai & Jain (2012) to be required for the invasion of brain endothelia and Rv0805 was found to be important for the invasion and survival within brain tissue in a murine model (Be et al., 2008). The genes shared by UM-CSF strains and *S. pneumoniae* are involved in the biosynthesis of pyrimidine (pyrG, Rv1699), purine (purA, Rv0357c) and pyridoxine (Rv2606c). Both purA (adenylosuccinate synthetase) and pyrG (CTP synthase) were associated with attenuated *S. pneumonia* replication during experimental meningitis (Molzen et al., 2011) and pyridoxine is important for the functioning of nerves. One homolog of *N. meningitidis* (Rv2457c) is a chaperone of the ATP-dependent CLP protease that is required for in vitro and in vivo growth of *Mtb* (Raju et al., 2012). The other homolog (Rv2397c) is a part of the ABC transporter complex involved in sulfate/thiosulfate import (Szklarczyk et al., 2015).

In summary, in our UM-CSF strains, we found large-scale and smaller genomic rearrangements, indels, gene truncations and micro-variants that we did not detect in our comparison respiratory strains. All eight CSF strains shared common protein globularity differences against H37Rv and common homologs of meningitis-associated proteins in *Mtb*, *S. pneumoniae* and *N. meningitidis*. However, we failed to identify features more apparently related to TB neurotropism or neurovirulence such as proteins involved with changes in brain vascular endothelium or extracellular matrix composition that could result in increased permeability of the blood-brain-barrier. This could be partly due to the abundance of PE/PPE proteins and other proteins with unknown function, among the traits we observed. Future investigations could reveal their involvement in CNS tropism and pathology.

We were also disappointed to find that many of the globularity changes and virulence factors in our CSF strains (including those from *S. pneumoniae* and *N. meningitidis*) were also present in our comparison respiratory strains. However, this finding is consistent with the observation by other workers that many virulence genes are conserved in non-pathogenic bacteria. All four *mce* operons in the genome of *Mtb* (Kumar, Bose & Brahmachari, 2003) have been found in both pathogenic and non-pathogenic mycobacteria (Haile et al., 2002; Chitale et al., 2001). CLP proteases on the whole, are common in many bacterial spp. (De Mot et al., 1999). The ABC transporter complex involved in sulfate/thiosulfate import is found in pathogens as well as environmental bacteria (Szklarczyk et al., 2015). Many designated virulence genes in *N. meningitidis* were also found to be present in nonpathogenic species such as *N. lactamica* (Snyder & Saunders, 2006). All these observations suggest that pathogenic bacteria have adapted their genomes from a free-lifestyle to the intracellular environment with minimal acquisition of exclusive virulence genes (Forrellad et al., 2013). Hence, by analogy, we can also hypothesize that CNS tropism in TB is not driven by the presence of specific genetic traits but by the expression of multiple virulence factors, probably elicited in response to host immune defences.

Nonetheless, the detection of *S. pneumoniae* and *N. meningitidis* gene orthologs in our UM-CSF strains raises speculations on the existence of a pan-bacterial mechanism of CNS infection. Further investigations on our observations might lead to new understanding and new strategies in the management of tuberculous as well as other bacterial CNS infections.

## Conclusion

Many genetic traits have been described for bacterial pathogens causing CNS infection. We detected large-scale rearrangements, short translocations, inversions, indels and nsSNPs in our CSF-derived strains, as well as protein globularity changes and orthologs of meningitis-associated genes previously reported in other neuropathogenic bacteria. Many of these features are, however, not CSF-specific or consistently present in all CSF strains. Hence, our findings suggest that neurotropism and neurovirulence in TBM is directed by the expression of multiple virulence factors selected by the interaction between pathogen and host immune responses rather than the presence of specific genetic traits.

## Supplemental Information

10.7717/peerj.2484/supp-1 Supplemental Information 1 Dotplots showing rearrangements in UM-CSF01 and UM-CSF05.

10.7717/peerj.2484/supp-2 Supplemental Information 2 Identifications of rearrangements in the genomes which were constructed by different algorithms.

10.7717/peerj.2484/supp-3 Supplemental Information 3 Identification of rearrangement in UM-CSF06.

10.7717/peerj.2484/supp-4 Supplemental Information 4 Identification of rearrangement in UM-CSF09.

10.7717/peerj.2484/supp-5 Supplemental Information 5 Identification of rearrangement in UM-CSF15.

10.7717/peerj.2484/supp-6 Supplemental Information 6 Identification of rearrangement in UM-CSF17.

10.7717/peerj.2484/supp-7 Supplemental Information 7 List of representative strains used for rearrangement analysis.

10.7717/peerj.2484/supp-8 Supplemental Information 8 List of sequencing reads used for microvariants analysis.

10.7717/peerj.2484/supp-9 Supplemental Information 9 List of genes within rearranged regions in UM-CSF01.

10.7717/peerj.2484/supp-10 Supplemental Information 10 List of affected genes within rearranged regions in UM-CSF05.

10.7717/peerj.2484/supp-11 Supplemental Information 11 List of affected genes within rearranged regions in UM-CSF06, UM-CSF09, UM-CSF15 and UM-CSF17.

10.7717/peerj.2484/supp-12 Supplemental Information 12 Non-synonymous variants specific in at least four UM-CSF strains.

10.7717/peerj.2484/supp-13 Supplemental Information 13 Enrichment Analysis on Sets of Proteins with Different Domain Structures.

10.7717/peerj.2484/supp-14 Supplemental Information 14 List of meningitis-associated factors in *M. tuberculosis*.

10.7717/peerj.2484/supp-15 Supplemental Information 15 List of meningitis-associated genes in *S. pnemoniae*.

10.7717/peerj.2484/supp-16 Supplemental Information 16 List of meningitis-associated factors in *N. meningitidis*.

10.7717/peerj.2484/supp-17 Supplemental Information 17 List of meningitis-associated factors in *E. coli*.

## Additional Information and Declarations

### DNA Deposition

The following information was supplied regarding the deposition of DNA sequences: The Whole Genome Shotgun project for UM-CSF01 and UM-CSF05 has been deposited at DDBJ/ENA/GenBank under the accessions LLXF00000000, LLXG00000000, respectively and that for UM-CSF04, UM-CSF06, UM-CSF08, UM-CSF09, UM-CSF15 and UM-CSF17 under the accessions LXGA00000000, LXGB00000000, LXGC00000000, LXGD00000000, LXGE00000000 and LXGF00000000, respectively.

## References

[ref-1] Abdallah AM, Verboom T, Hannes F, Safi M, Strong M, Eisenberg D, Musters RJP, Vandenbroucke-Grauls CMJE, Appelmelk BJ, Luirink J, Bitter W (2006). A specific secretion system mediates PPE41 transport in pathogenic mycobacteria. Molecular Microbiology.

[ref-3] Angiuoli SV, Salzberg SL (2011). Mugsy: fast multiple alignment of closely related whole genomes. Bioinformatics.

[ref-4] Arvanitakis Z1, Long RL, Hershfield ES, Manfreda J, Kabani A, Kunimoto D, Power C (1998). *M. tuberculosis* molecular variation in CNS infection: evidence for strain-dependent neurovirulence. Neurology.

[ref-5] Av-Gay Y, Everett M (2000). The eukaryotic-like Ser/Thr protein kinases of *Mycobacterium tuberculosis*. Trends in Microbiology.

[ref-6] Be NA, Bishai WR, Jain SK (2012). Role of *Mycobacterium tuberculosis pknD* in the pathogenesis of central nervous system tuberculosis. BMC Microbiology.

[ref-7] Be NA, Lamichhane G, Grosset J, Tyagi S, Cheng Q-J, Kim KS, Bishai WR, Jain SK (2008). Murine model to study the invasion and survival of *Mycobacterium tuberculosis* in the central nervous system. Journal of Infectious Diseases.

[ref-8] Besemer J, Lomsadze A, Borodovsky M (2001). GeneMarkS: a self-training method for prediction of gene starts in microbial genomes. Implications for finding sequence motifs in regulatory regions. Nucleic Acids Research.

[ref-9] Bidstrup C, Andersen PH, Skinhøj P, Andersen AB (2002). Tuberculous meningitis in a country with a low incidence of tuberculosis: still a serious disease and a diagnostic challenge. Scandinavian Journal of Infectious Diseases.

[ref-10] Boetzer M, Henkel CV, Jansen HJ, Butler D, Pirovano W (2011). Scaffolding pre-assembled contigs using SSPACE. Bioinformatics.

[ref-11] Campo M, Randhawa AK, Dunstan S, Farrar J, Caws M, Bang ND, Lan NN, Hong Chau TT, Horne DJ, Thuong NT, Thwaites GE, Hawn TR (2015). Common polymorphisms in the CD43 gene region are associated with tuberculosis disease and mortality. American Journal of Respiratory Cell and Molecular Biology.

[ref-12] Caws M, Thwaites G, Dunstan S, Hawn TR, Lan NTN, Thuong NTN, Stepniewska K, Huyen MNT, Bang ND, Loc TH, Gagneux S, van Soolingen D, Kremer K, van der Sande M, Small P, Anh PTH, Chinh NTN, Quy HT, Duyen NTN, Tho DQ, Hieu NTN, Torok E, Hien TT, Dung NH, Nhu NTN, Duy PM, van Vinh Chau N, Farrar J (2008). The influence of host and bacterial genotype on the development of disseminated disease with *Mycobacterium tuberculosis*. PLoS Pathogens.

[ref-13] Caws M, Thwaites G, Stepniewska K, Nguyen TN, Nguyen TH, Nguyen TP, Mai NT, Phan MD, Tran HL, Tran TH, van Soolingen D, Kremer K, Nguyen VV, Nguyen TC, Farrar J (2006). Beijing genotype of *Mycobacterium tuberculosis* is significantly associated with human immunodeficiency virus infection and multidrug resistance in cases of tuberculous meningitis. Journal of Clinical Microbiology.

[ref-14] Capriotti E, Fariselli P, Casadio R (2005). I-Mutant2.0: predicting stability changes upon mutation from the protein sequence or structure. Nucleic Acids Research.

[ref-15] Chitale S, Ehrt S, Kawamura I, Fujimura T, Shimono N, Anand N, Lu S, Cohen-Gould L, Riley LW (2001). Recombinant *Mycobacterium tuberculosis* protein associated with mammalian cell entry. Cellular Microbiology.

[ref-16] Cingolani P, Platts A, Wang LL, Coon M, Nguyen T, Wang L, Land SJ, Lu X, Ruden DM (2012). A program for annotating and predicting the effects of single nucleotide polymorphisms, SnpEff: SNPs in the genome of *Drosophila melanogaster* strain w1118; iso-2; iso-3. Fly.

[ref-17] Cole ST, Brosch R, Parkhill J, Garnier T, Churcher C, Harris D, Gordon SV, Eiglmeier K, Gas S, Barry CE, Tekaia F, Badcock K, Basham D, Brown D, Chillingworth T, Connor R, Davies R, Devlin K, Feltwell T, Gentles S, Hamlin N, Holroyd S, Hornsby T, Jagels K, Krogh A, McLean J, Moule S, Murphy L, Oliver K, Osborne J, Quail M-A, Rajandream M-A, Rogers J, Rutter S, Seeger K, Skelton J, Squares R, Squares S, Sulston JE, Taylor K, Whitehead S, Barrell BG (1998). Deciphering the biology of *Mycobacterium tuberculosis* from the complete genome sequence. Nature.

[ref-18] Darling ACE, Mau B, Blattner FR, Perna NT (2004). Mauve: multiple alignment of conserved genomic sequence with rearrangements. Genome Research.

[ref-19] Darwin KH, Nathan CF (2005). Role for nucleotide excision repair in virulence of *Mycobacterium tuberculosis*. Infection and Immunity.

[ref-20] Davidsen T, Tuven HK, Bjørås M, Rødland EA, Tønjum T (2007). Genetic interactions of DNA repair pathways in the pathogen *Neisseria meningitides*. Journal of Bacteriology.

[ref-21] De Mot R, Nagy I, Walz J, Baumeister W (1999). Proteasomes and other self-compartmentalizing proteases in prokaryotes. Trends in Microbiology.

[ref-22] de Viedma DG, Marín M, Andrés S, Lorenzo G, Ruiz-Serrano MJ, Bouza E (2006). Complex clonal features in an *Mycobacterium tuberculosis* infection in a two-year-old child. The Pediatric Infectious Disease Journal.

[ref-23] Dennis G, Sherman BT, Hosack DA, Yang J, Gao W, Lane HC, Lempicki RA (2003). DAVID: database for annotation, visualization, and integrated discovery. Genome Biology.

[ref-24] Dunker AK, Lawson JD, Brown CJ, Williams RM, Romero P, Oh JS, Oldfield CJ, Campen AM, Ratliff CM, Hipps KW, Ausio J, Nissen MS, Reeves R, Kang CH, Kissinger CR, Bailey RW, Griswold MD, Chiu W, Garner EC, Obradovic Z (2001). Intrinsically disordered protein. Journal of Molecular Graphics and Modelling.

[ref-25] Elmas ÖN, Akinci A, Bilir P (2011). Tuberculous meningitis associated with diabetic ketoacidosis. Journal of Clinical Research in Pediatric Endocrinology.

[ref-26] Erhabor GE, Adewole OO, Ogunlade O (2006). A five year review of tuberculosis mortality amongst hospitalized patients in Ile-Ife. Indian Journal of Chest Disease and Allied Science.

[ref-27] Farooqui AA, Horrocks LA, Farooqui T (2000). Glycerophospholipids in brain: their metabolism, incorporation into membranes, functions, and involvement in neurological disorders. Chemistry and Physics of Lipids.

[ref-28] Fleischmann RD, Alland D, Eisen JA, Carpenter L, White O, Peterson J, DeBoy R, Dodson R, Gwinn M, Haft D, Hickey E, Kolonay JF, Nelson WC, Umayam LA, Ermolaeva M, Salzberg SL, Delcher A, Utterback T, Weidman J, Khouri H, Gill J, Mikula A, Bishai W, Jacobs WR, Venter JC, Fraser CM (2002). Whole-genome comparison of *Mycobacterium tuberculosis* clinical and laboratory strains. Journal of Bacteriology.

[ref-29] Forrellad MA, Klepp LI, Gioffré A, Sabio y García J, Morbidoni HR, Santangelo MDLP, Cataldi AA, Bigi F (2013). Virulence factors of the *Mycobacterium tuberculosis* complex. Virulence.

[ref-30] García-Gómez E, González-Pedrajo B, Camacho-Arroyo I (2013). Role of sex steroid hormones in bacterial-host interactions. BioMed Research International.

[ref-32] Greninger AL, Langelier C, Cunningham G, Keh C, Melgar M, Chiu CY, Miller S (2015). Two rapidly growing mycobacterial species isolated from a brain abscess: first whole-genome sequences of *Mycobacterium immunogenum* and *Mycobacterium llatzerense*. Journal of Clinical Microbiology.

[ref-33] Haile Y, Caugant DA, Bjune G, Wiker HG (2002). *Mycobacterium tuberculosis* mammalian cell entry operon (*mce*) homologs in *Mycobacterium* other than tuberculosis (MOTT). FEMS Immunology & Medical Microbiology.

[ref-34] Haldar S, Sankhyan N, Sharma N, Bansal A, Jain V, Gupta VK, Juneja M, Mishra D, Kapil A, Singh UB, Gulati S, Kalra V, Tyagi JS (2012). Detection of *Mycobacterium tuberculosis GlcB* or *HspX* antigens or *devR* DNA impacts the rapid diagnosis of tuberculous meningitis in children. PLoS ONE.

[ref-35] Hao W, Ma JH, Warren K, Tsang RSW, Low DE, Jamieson FB, Alexander DC (2011). Extensive genomic variation within clonal complexes of *Neisseria meningitidis*. Genome Biology and Evolution.

[ref-36] Hawn TR, Dunstan SJ, Thwaites GE, Simmons CP, Thuong NTN, Lan NTN, Quy HT, Chau TTH, Hieu NTN, Rodrigues S, Janer M, Zhao LP, Hien TTH, Farrar JJ, Aderem A (2006). A polymorphism in Toll-interleukin 1 receptor domain containing adaptor protein is associated with susceptibility to meningeal tuberculosis. Journal of Infectious Diseases.

[ref-57] Hernandez Pando R, Aguilar D, Cohen I, Guerrero M, Ribon W, Acosta P, Orozco H, Marquina B, Salinas C, Rembao D, Espitia C (2010). Specific bacterial genotypes of *Mycobacterium tuberculosis* cause extensive dissemination and brain infection in an experimental model. Tuberculosis.

[ref-37] Hesseling AC, Marais BJ, Kirchner HL, Mandalakas AM, Brittle W, Victor TC, Warren RM, Schaaf HS (2010). Mycobacterial genotype is associated with disease phenotype in children. The International Journal of Tuberculosis and Lung Disease.

[ref-38] Hoal-Van Helden EG, Epstein J, Victor TC, Hon D, Lewis L-A, Beyers N, Zurakowski D, Ezekowitz RAB, Van Helden PD (1999). Mannose-binding protein B allele confers protection against tuberculous meningitis. Pediatric Research.

[ref-39] Huang S-H, Jong AY (2001). Cellular mechanisms of microbial proteins contributing to invasion of the blood-brain barrier. Cellular Microbiology.

[ref-40] Huang S-H, Wan Z-S, Chen Y-H, Jong AY, Kim KS (2001). Further characterization of *Escherichia coli* brain microvascular endothelial cell invasion gene *ibeA* by deletion, complementation, and protein expression. Journal of Infectious Diseases.

[ref-41] Ige OM, Sogaolu OM, Ogunlade OA (2005). Pattern of presentation of tuberculosis and the hospital prevalence of tuberculosis and HIVco-infection in University College Hospital, Ibadan: a review of five years (1998–2002). African Journal of Medical and Health Sciences.

[ref-42] Jain SK, Paul-Satyaseela M, Lamichhane G, Kim KS, Bishai WR (2006). *Mycobacterium tuberculosis* invasion and traversal across an in vitro human blood-brain barrier as a pathogenic mechanism for central nervous system tuberculosis. Journal of Infectious Diseases.

[ref-43] Keane J, Gershon S, Wise RP, Mirabile-Levens E, Kasznica J, Schwieterman WD, Siegel JN, Braun MM (2001). Tuberculosis associated with infliximab, a tumor necrosis factor α–neutralizing agent. New England Journal of Medicine.

[ref-44] Krumsiek J, Arnold R, Rattei T (2007). Gepard: a rapid and sensitive tool for creating dotplots on genome scale. Bioinformatics.

[ref-45] Kumar A, Bose M, Brahmachari V (2003). Analysis of expression profile of mammalian cell entry (*mce*) operons of *Mycobacterium tuberculosis*. Infection and Immunity.

[ref-46] Lechner M, Findeiss S, Steiner L, Marz M, Stadler PF, Prohaska SJ (2011). Proteinortho: detection of (Co-)orthologs in large-scale analysis. BMC Bioinformatics.

[ref-47] Li H, Handsaker B, Wysoker A, Fennell T, Ruan J, Homer N, Marth G, Abecasis G, Durbin R, 1000 Genome Project Data Processing Subgroup (2009). The sequence alignment/map format and SAMtools. Bioinformatics.

[ref-48] Linding R, Russell RB, Neduva V, Gibson TJ (2003). GlobPlot: exploring protein sequences for globularity and disorder. Nucleic Acids Research.

[ref-49] Mahdi LK, Wang H, Van der Hoek MB, Paton JC, Ogunniyi AD (2012). Identification of a novel pneumococcal vaccine antigen preferentially expressed during meningitis in mice. Journal of Clinical Investigation.

[ref-50] Molzen TE, Burghout P, Bootsma HJ, Brandt CT, van der Gaast-de Jongh CE, Eleveld MJ, Verbeek MM, Frimodt-Møller N, Østergaard C, Hermans PWM (2011). Genome-wide identification of *Streptococcus pneumoniae* genes essential for bacterial replication during experimental meningitis. Infection and Immunity.

[ref-52] Neyrolles O, Quintana-Murci L (2009). Sexual inequality in tuberculosis. PLoS Medicine.

[ref-53] NIMHANS (2010). http://www.nimhans.kar.nic.in/neuromicrobiology/default.htm.

[ref-54] Norton JP, Mulvey MA (2012). Toxin-antitoxin systems are important for niche-specific colonization and stress resistance of uropathogenic *Escherichia coli*. PLoS Pathogens.

[ref-55] Okumura K, Kato M, Kirikae T, Kayano M, Miyoshi-Akiyama T (2015). Construction of a virtual *Mycobacterium tuberculosis* consensus genome and its application to data from a next generation sequencer. BMC Genomics.

[ref-56] Orihuela CJ, Radin JN, Sublett JE, Gao G, Kaushal D, Tuomanen EI (2004). Microarray analysis of pneumococcal gene expression during invasive disease. Infection and Immunity.

[ref-58] Peng Y, Leung HCM, Yiu SM, Chin FYL (2012). IDBA-UD: a de novo assembler for single-cell and metagenomic sequencing data with highly uneven depth. Bioinformatics.

[ref-59] Pethe K, Alonso S, Biet F, Delogu G, Brennan MJ, Locht C, Menozzi FD (2001). The heparin-binding haemagglutinin of *M. tuberculosis* is required for extrapulmonary dissemination. Nature.

[ref-60] Pieters J, Gatfield J (2002). Hijacking the host: survival of pathogenic mycobacteria inside macrophages. Trends in Microbiology.

[ref-61] Pouttu R, Puustinen T, Virkola R, Hacker J, Klemm P, Korhonen TK (1999). Amino acid residue Ala-62 in the *FimH* fimbrial adhesion is critical for the adhesiveness of meningitis-associated *Escherichia coli* to collagens. Molecular Microbiology.

[ref-62] Raju RM, Unnikrishnan M, Rubin DHF, Krishnamoorthy V, Kandror O, Akopian TN, Goldberg AL, Rubin EJ (2012). *Mycobacterium tuberculosis* ClpP1 and ClpP2 function together in protein degradation and are required for viability *in vitro* and during infection. PLoS Pathogens.

[ref-63] Ramage HR, Connolly LE, Cox JS (2009). Comprehensive functional analysis of *Mycobacterium tuberculosis* toxin-antitoxin systems: implications for pathogenesis, stress responses, and evolution. PLoS Genetics.

[ref-64] Reed MB, Domenech P, Manca C, Su H, Barczak AK, Kreiswirth BN, Kaplan G, Barry CE III (2004). A glycolipid of hypervirulent tuberculosis strains that inhibits the innate immune response. Nature.

[ref-65] Sambrook J, Fritsch EF, Maniatis T (1989). Molecular Cloning. A Laboratory Manual.

[ref-67] Shitikov EA, Bespyatykh JA, Ischenko DS, Alexeev DG, Karpova IY, Kostryukova ES, Isaeva YD, Nosova EY, Mokrousov IV, Vyazovaya AA, Narvskaya OV, Vishnevsky BI, Otten TF, Zhuravlev VY, Yablonsky PK, Ilina EN, Govorun VM (2014). Unusual large-scale chromosomal rearrangements in *Mycobacterium tuberculosis* Beijing B0/W148 cluster isolates. PLoS ONE.

[ref-68] Shoichet BK, Baase WA, Kuroki R, Matthews BW (1995). A relationship between protein stability and protein function. Proceedings of the National Academy of Sciences of the United States of America.

[ref-69] Smith I (2003). *Mycobacterium tuberculosis* pathogenesis and molecular determinants of virulence. Clinical Microbiology Reviews.

[ref-70] Song H, Sandie R, Wang Y, Andrade-Navarro MA, Niederweis M (2008). Identification of outer membrane proteins of *Mycobacterium tuberculosis*. Tuberculosis.

[ref-71] Szklarczyk D, Franceschini A, Wyder S, Forslund K, Heller D, Huerta-Cepas J, Simonovic M, Roth A, Santos A, Tsafou KP, Kuhn M, Bork P, Jensen LJ, von Mering C (2015). STRING v10: protein–protein interaction networks, integrated over the tree of life. Nucleic Acids Research.

[ref-72] Studer RA, Dessailly BH, Orengo CA (2013). Residue mutations and their impact on protein structure and function: detecting beneficial and pathogenic changes. Biochemical Journal.

[ref-73] Snyder LA, Saunders NJ (2006). The majority of genes in the pathogenic *Neisseria* species are present in non-pathogenic *Neisseria lactamica*, including those designated as ‘virulence genes’. BMC Genomics.

[ref-74] Talarico S, Donald Cave M, Foxman B, Marrs CF, Zhang L, Bates JH, Yang Z (2007). Association of *Mycobacterium tuberculosis* PE_PGRS33 polymorphism with clinical and epidemiological characteristics. Tuberculosis.

[ref-75] Tokuriki N, Stricher F, Serrano L, Tawfik DS (2008). How protein stability and new functions trade off. PLoS Computational Biology.

[ref-76] Tsenova L, Ellison E, Harbacheuski R, Moreira AL, Kurepina N, Reed MB, Mathema B, Barry CE III, Kaplan G (2005). Virulence of selected *Mycobacterium tuberculosis* clinical isolates in the rabbit model of meningitis is dependent on phenolic glycolipid produced by the bacilli. Journal of Infectious Diseases.

[ref-77] Tsolaki AG, Hirsh AE, DeRiemer K, Enciso JA, Wong MZ, Hannan M, Goguet de la Salmoniere YOL, Aman K, Kato-Maeda M, Small PM (2004). Functional and evolutionary genomics of *Mycobacterium tuberculosis*: insights from genomic deletions in 100 strains. Proceedings of the National Academy of Sciences of the United States of America.

[ref-78] Vinnard C, Macgregor RR (2009). Tuberculous meningitis in HIV-infected individuals. Current HIV/AIDS Reports.

[ref-79] Virji M, Makepeace K, Ferguson DJP, Achtman M, Moxon ER (1993). Meningococcal Opa and Opc proteins: their role in colonization and invasion of human epithelial and endothelial cells. Molecular Microbiology.

[ref-80] Wang X, Minasov G, Shoichet BK (2002). Evolution of an antibiotic resistance enzyme constrained by stability and activity trade-offs. Journal of Molecular Biology.

[ref-81] Yao Y, Xie Y, Kim KS (2006). Genomic comparison of *Escherichia coli* K1 strains isolated from the cerebrospinal fluid of patients with meningitis. Journal of Infectious Diseases.

