# Supplementary figures and images for: Chromosomal rearrangements and protein globularity changes in Mycobacterium tuberculosis isolates from cerebrospinal fluid
Source: PeerJ. 2016 Sep 21;4:e2484. doi: 10.7717/peerj.2484 (PMC5036109; doi:10.7717/peerj.2484)

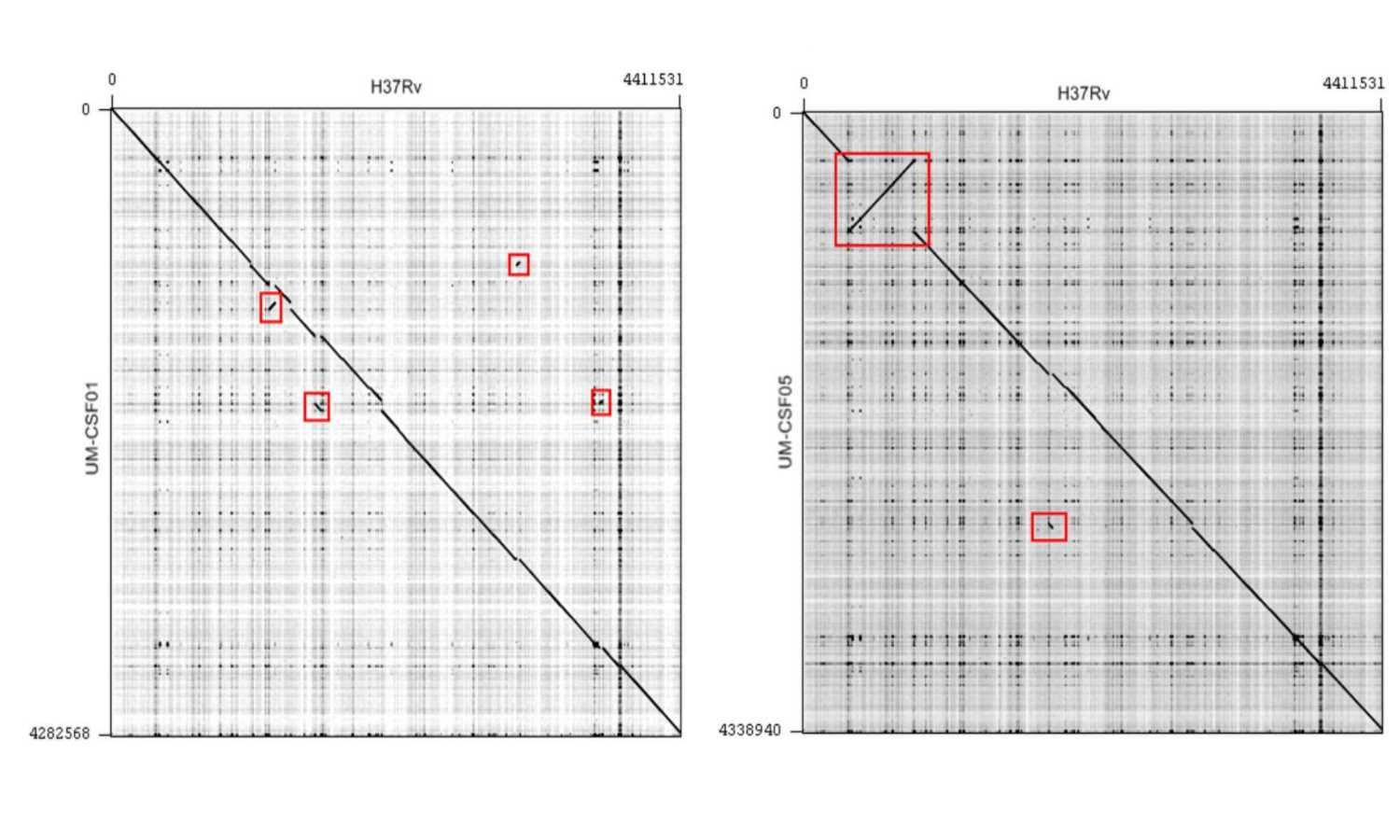

Supplement: Supplemental Information 1 [file peerj-04-2484-s001.png]

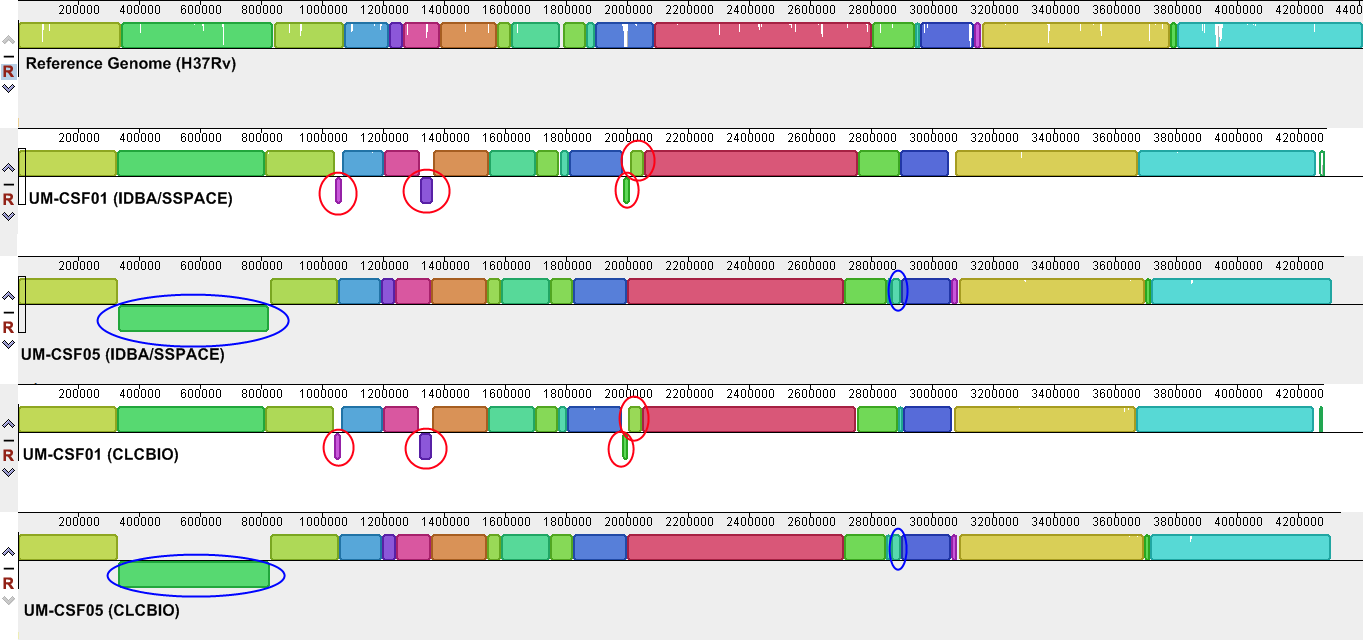

Supplement: Supplemental Information 2 [file peerj-04-2484-s002.png]

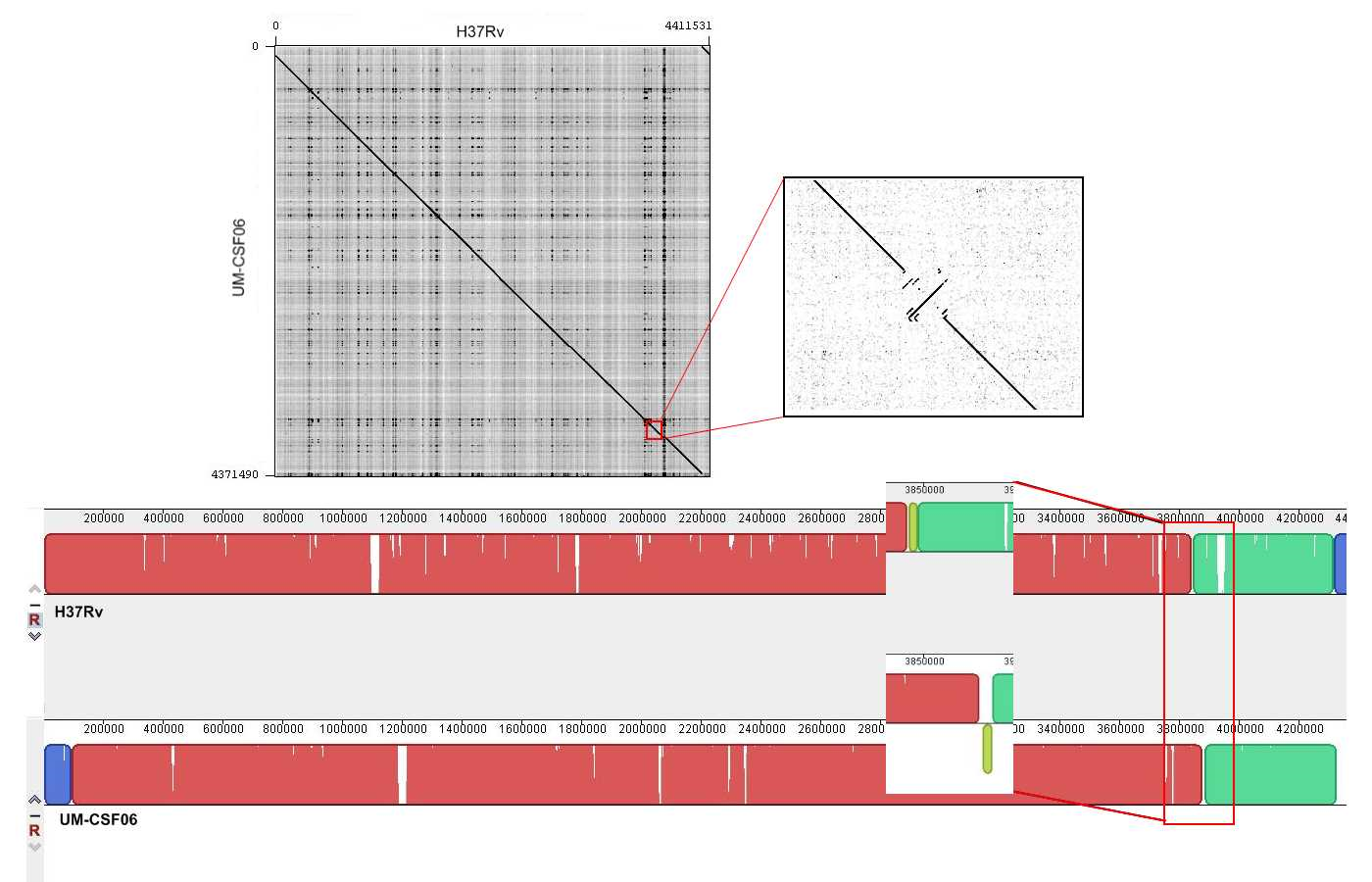

Supplement: Supplemental Information 3 [file peerj-04-2484-s003.png]

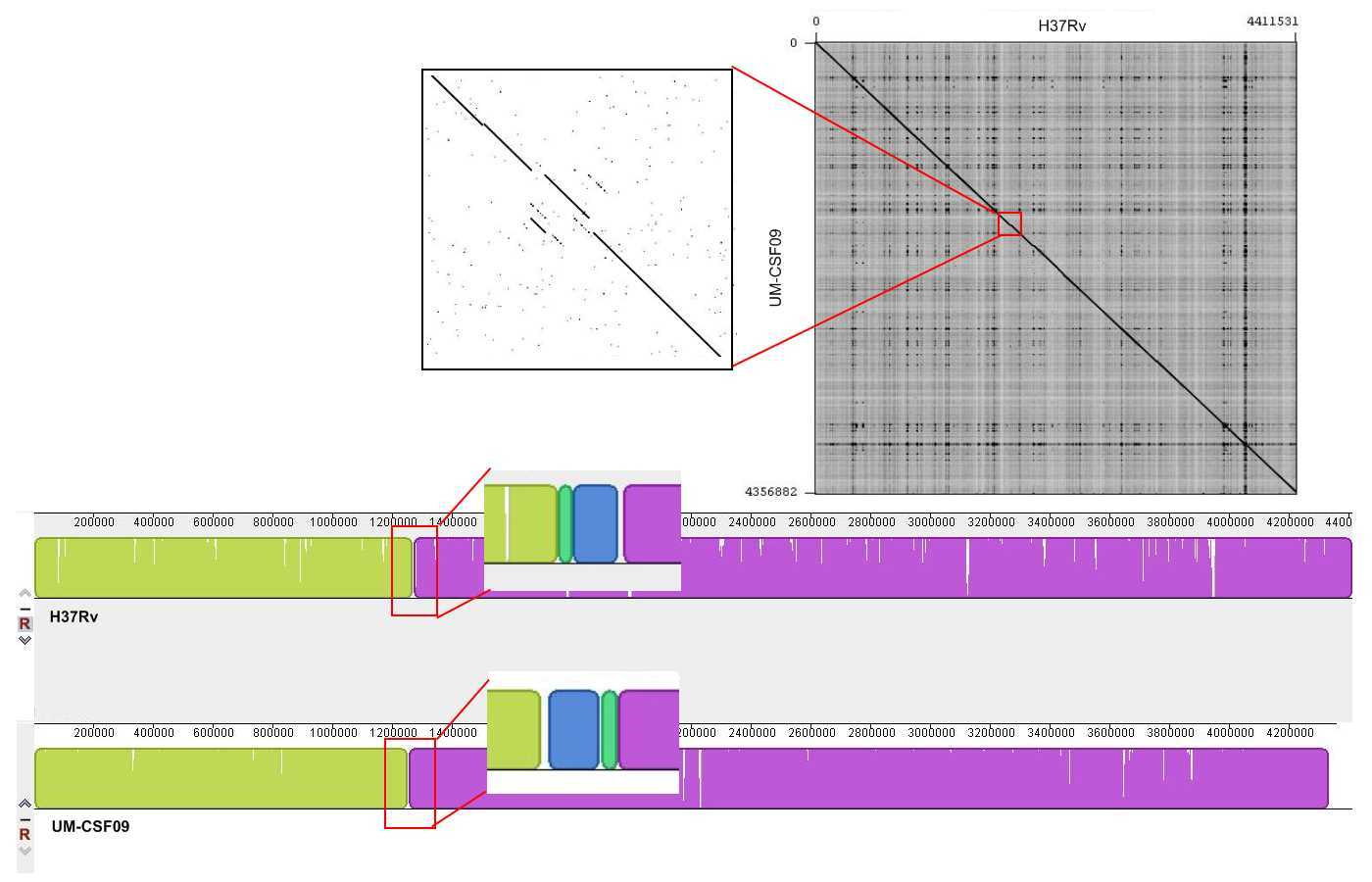

Supplement: Supplemental Information 4 [file peerj-04-2484-s004.png]

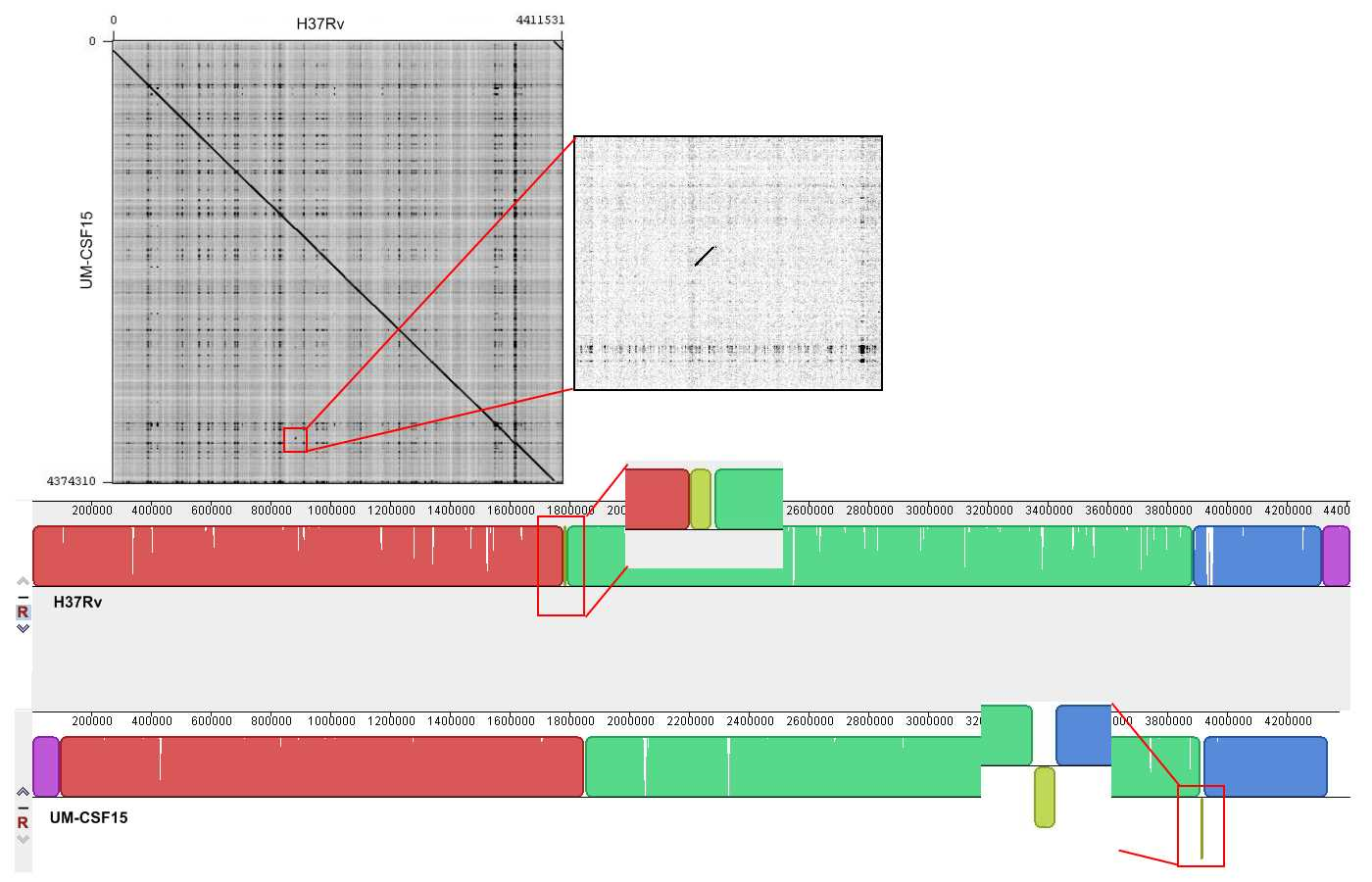

Supplement: Supplemental Information 5 [file peerj-04-2484-s005.png]

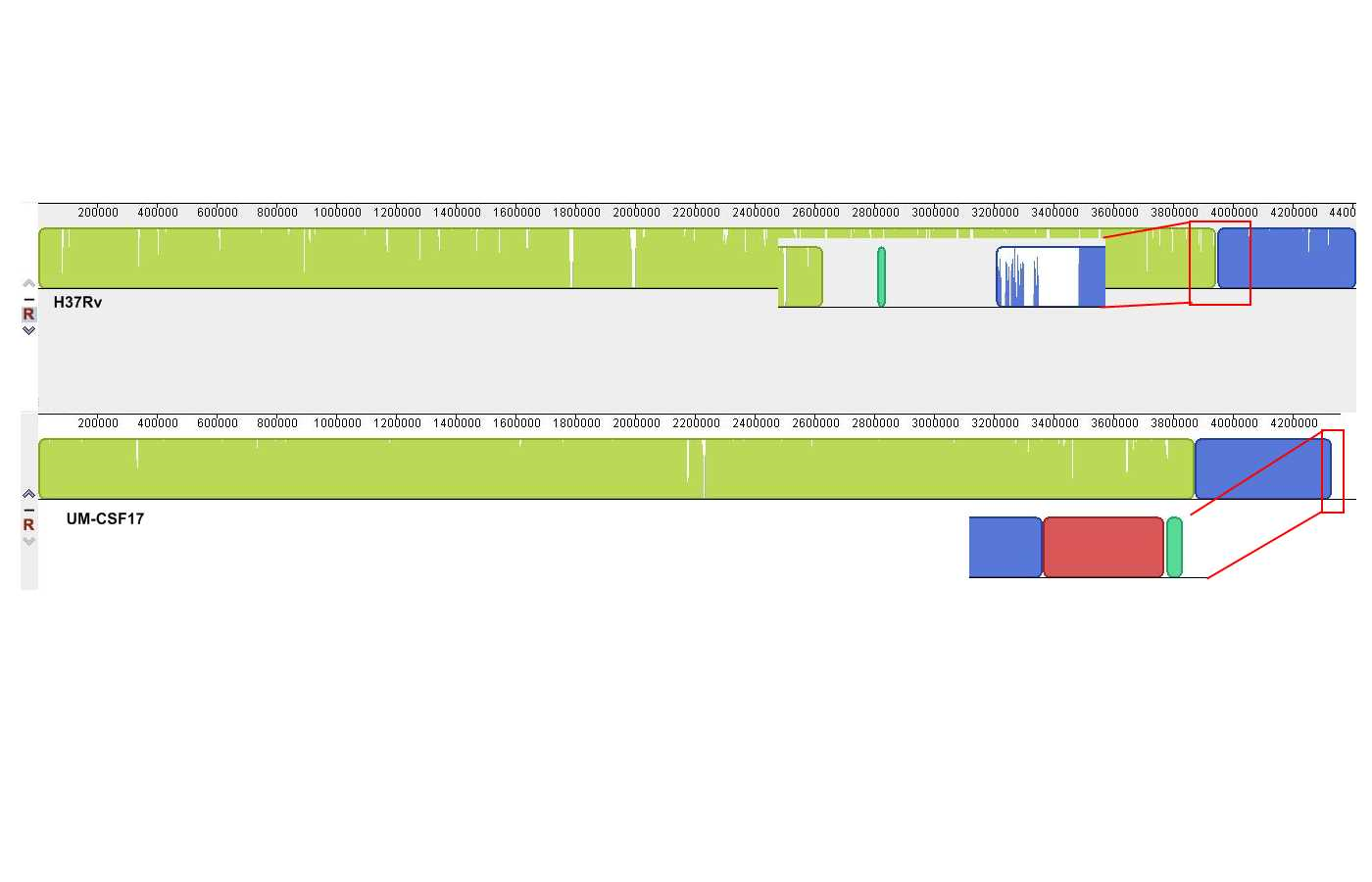

Supplement: Supplemental Information 6 [file peerj-04-2484-s006.png]
