# Supplementary material for: Chromosomal rearrangements and protein globularity changes in Mycobacterium tuberculosis isolates from cerebrospinal fluid
Source: PeerJ. 2016 Sep 21;4:e2484. doi: 10.7717/peerj.2484 (PMC5036109; doi:10.7717/peerj.2484)
Supplement: Supplemental Information 7 [file peerj-04-2484-s007.pdf]

NCBI Group ID	Representative Strain	Accession No.
1	H37Rv	NC_000962.3
2	0B235DS	CP008962.1
3	HN878	NZ_ADNF00000000.1
4	S96-129	NZ_AEGB00000000.1
5	98-R604 INH-RIF-EM	NZ_ABVM00000000.1
6	W-148	NZ_ACSX00000000.1
7	2074CJ	NZ_JKBN00000000.1
8	2230BH	NZ_JKBM00000000.1
9	M1734	NZ_JKMF00000000.1
10	TB_RSA57	NZ_JKVI00000000.1
11	Mtb194	NZ_AUNH00000000.1
12	OSDD071	NZ_AHHX00000000.1
13	KT-0187	NZ_JUFA00000000.1
14	Aethiop_vetus_233	CEGA00000000.1
15	A70011_4	CQFD00000000.1
16	A70136	CQFB00000000.1
17	A70448	CQFF00000000.1
