# Supplementary material for: Chromosomal rearrangements and protein globularity changes in Mycobacterium tuberculosis isolates from cerebrospinal fluid
Source: PeerJ. 2016 Sep 21;4:e2484. doi: 10.7717/peerj.2484 (PMC5036109; doi:10.7717/peerj.2484)
Supplement: Supplemental Information 8 [file peerj-04-2484-s008.pdf]

Strains	SRA Number	Platform	Location
ERX009495	ERR023195	Illumina Genome Analyzer II	Edinburgh
ERX157998	ERR181888	Illumina HiSeq 2000	Malawi
ERX509474	ERR550405	Illumina MiSeq	Ireland
ERX662243	ERR718321	Illumina HiSeq 2000	Thailand
ERX662267	ERR718345	Illumina HiSeq 2000	Thailand
ERX662394	ERR718472	Illumina HiSeq 2000	Thailand
ERX680508	ERR736817	Illumina HiSeq 2000	Karonga Malawi
ERX680519	ERR736828	Illumina HiSeq 2000	Karonga Malawi
ERX701819	ERR757189	Illumina MiSeq	Argentina
M	ERR764904	Illumina MiSeq	Argentina
SRX473357	SRR1172814	Illumina HiSeq 2000	Stanger Hospital
SRX473469	SRR1172957	Illumina HiSeq 2000	Church of Scotland
SRX473507	SRR1173000	Illumina HiSeq 2000	Sihleza Clinic
SRX473631	SRR1173185	Illumina HiSeq 2000	Richard's Bay Clinic
SRX473638	SRR1173193	Illumina HiSeq 2000	Dundee Hospital
SRX473664	SRR1173223	Illumina HiSeq 2000	Thulasizwe Hopsital
SRX473782	SRR1173375	Illumina HiSeq 2000	St Margaret's Hospital
SRX473864	SRR1173482	Illumina HiSeq 2000	Doris Goodwin Hospital
SRX473807	SRR1173560	Illumina HiSeq 2000	Chwezi Clinic
SRX474483	SRR1174317	Illumina HiSeq 2000	Uganda
SRX475357	SRR1175470	Illumina HiSeq 2000	Romania
SRX479642	SRR1180449	Illumina HiSeq 2000	India: Tiruvallur
SRX480066	SRR1181090	Illumina HiSeq 2000	Buenaventura/Valle del Cauca/Colombia
SRX480028	SRR1184026	Illumina HiSeq 2000	Durban Chest Clinic
SRX481760	SRR1184322	Illumina HiSeq 2000	King Edward VIII Hospital
SRX481812	SRR1184378	Illumina HiSeq 2000	Thulasizwe Hospital
SRX493314	SRR1196472	Illumina HiSeq 2000	Ngwelezana Hospital
SRX493320	SRR1196478	Illumina HiSeq 2000	Richard's Bay Clinic
SRX493351	SRR1196511	Illumina HiSeq 2000	Goodwins Clinic
SRX493355	SRR1196515	Illumina HiSeq 2000	FOSA Hospital
SRX493361	SRR1196523	Illumina HiSeq 2000	Manguzi Hospital
SRX493377	SRR1196539	Illumina HiSeq 2000	Edendale Hospital
SRX493382	SRR1196544	Illumina HiSeq 2000	Dundee Hospital
SRX493388	SRR1196551	Illumina HiSeq 2000	Catherine Booth
SRX493397	SRR1196563	Illumina HiSeq 2000	Shallcross Clinic

SRX493410	SRR1196576	Illumina HiSeq 2000	Chwezi Clinic
SRX493412	SRR1196580	Illumina HiSeq 2000	M3 TB Hospital
SRX493417	SRR1196586	Illumina HiSeq 2000	FOSA Clinic
SRX493473	SRR1196677	Illumina HiSeq 2000	Westville Prison
SRX493481	SRR1196685	Illumina HiSeq 2000	Mpumuza Clinic
SRX493499	SRR1196702	Illumina HiSeq 2000	Siloah Clinic
SRX493519	SRR1196723	Illumina HiSeq 2000	King Dinuzulu Hospital
SRX493557	SRR1196768	Illumina HiSeq 2000	King Dinuzulu Hospital
SRX648745	SRR1510041	Illumina HiSeq 2500	Manila
SRX648759	SRR1510055	Illumina MiSeq	Manila
SRX648774	SRR1510070	Illumina HiSeq 2500	Manila
SRX691156	SRR1564305	Illumina HiSeq 2000	Thailand
SRX699671	SRR1573680	Illumina MiSeq	Canada Ontario
SRX699698	SRR1573707	Illumina MiSeq	Canada Ontario
SRX691501	SRR1595970	Illumina HiSeq 2000	Thailand
MT-578	SRR1640752	Illumina MiSeq	Montreal Hospital
SRX816244	SRR1722712	Illumina HiSeq 2000	Russian Academy of Sciences
SRX816309	SRR1722867	Illumina HiSeq 2000	Russian Academy of Sciences
SRX347313	SRR974840	Illumina HiSeq 2000	South Africa: Western Cape
SRX347319	SRR974846	Illumina HiSeq 2000	Netherlands: Haarlem
SRX347317	SRR974849	Illumina HiSeq 2000	China
