# Supplementary material for: Chromosomal rearrangements and protein globularity changes in Mycobacterium tuberculosis isolates from cerebrospinal fluid
Source: PeerJ. 2016 Sep 21;4:e2484. doi: 10.7717/peerj.2484 (PMC5036109; doi:10.7717/peerj.2484)
Supplement: Supplemental Information 9 [file peerj-04-2484-s009.pdf]

Strand	Location	Gene	Gene Description
+	1211560..1213863	Rv1087	PE-PGRS family protein PE_PGRS21
+	1214040..1214360	Rv1087A	hypothetical protein
+	1214513..1214947	Rv1088	PE family protein PE9
+	<1214769..1215131	Rv1089	PE family protein PE10
+	1215517..1215621	Rv1089A	Probable cellulase CelA2a (endo-1,4-beta-glucanase) (endoglucanase) (carboxymethylcellulase)
+	1215599..1216054	Rv1090	Probable cellulase CelA2b (endo-1,4-beta-glucanase) (endoglucanase) (carboxymethylcellulase)
+	1216469..1219030	Rv1091	PE-PGRS family protein PE_PGRS22
-	1219248..1220186	Rv1092c	Probable pantothenate kinase CoaA (pantothenic acid kinase)
-	1220388..1220487	RVnc0034	Putative small regulatory RNA
+	1220574..1221854	Rv1093	Serine hydroxymethyltransferase 1 GlyA1
+	1221959..1222786	Rv1094	Possible acyl-[acyl-carrier protein] desaturase DesA2 (acyl-[ACP] desaturase) (stearoyl-ACP desaturase)
+	1222997..1224298	Rv1095	Probable PHOH-like protein PhoH2 (phosphate starvation-inducible protein PSIH)
+	1224385..1225260	Rv1096	Possible glycosyl hydrolase
-	1225263..1226144	Rv1097c	Probable membrane glycine and proline rich protein
-	1226141..1227565	Rv1098c	Probable fumarase Fum (fumarate hydratase)
-	1227596..1228684	Rv1099c	Fructose 1,6-bisphosphatase GlpX
+	1228683..1229384	Rv1100	hypothetical protein
-	1229391..1230548	Rv1101c	hypothetical protein
-	1230660..1230971	Rv1102c	Toxin MazF3
-	1230971..1231291	Rv1103c	Possible antitoxin MazE3
+	1231301..1231990	Rv1104	Possible para-nitrobenzyl esterase (fragment)
+	1232311..1232826	Rv1105	Possible para-nitrobenzyl esterase (fragment)
-	1232844..1233956	Rv1106c	3-beta-hydroxysteroid dehydrogenase
-	1233966..1234223	Rv1107c	Probable exodeoxyribonuclease VII (small subunit) XseB (exonuclease VII small subunit)
-	1234213..1235460	Rv1108c	Probable exodeoxyribonuclease VII (large subunit) XseA (exonuclease VII large subunit)

-	1235457..1236095	Rv1109c	hypothetical protein
+	1236185..1237192	Rv1110	Probable LYTB-related protein LytB2
-	1237209..1238192	Rv1111c	hypothetical protein
+	1238255..1239328	Rv1112	Probable GTP binding protein
+	1239416..1239613	Rv1113	Possible antitoxin VapB32
+	1239610..1239984	Rv1114	Possible toxin VapC32 Contains PIN domain
+	1240187..1240885	Rv1115	Possible exported protein
+	1241003..1241188	Rv1116	Hypothetical protein
-	1241115..1241390	Rv1116A	Conserved hypothetical protein (fragment)
+	1241633..1241956	Rv1117	hypothetical protein
-	1241971..1242831	Rv1118c	hypothetical protein
-	1242864..1243013	Rv1119c	Hypothetical protein
-	1243010..1243504	Rv1120c	hypothetical protein
+	1243707..1245107	Rv1121	Probable glucose-6-phosphate 1-dehydrogenase Zwf1 (G6PD)
+	1245129..1246151	Rv1122	Probable 6-phosphogluconate dehydrogenase,decarboxylating Gnd2
-	1246144..1247052	Rv1123c	Possible peroxidase BpoB (non-haem peroxidase)
+	1247127..1248077	Rv1124	Probable epoxide hydrolase EphC (epoxide hydratase)
+	1248082..1249326	Rv1125	hypothetical protein
-	1249330..1249935	Rv1126c	hypothetical protein
-	1249932..1251404	Rv1127c	Probable pyruvate, phosphate dikinase PpdK
-	1251617..1252972	Rv1128c	hypothetical protein
-	1253074..1254534	Rv1129c	Probable transcriptional regulator protein
+	1254555..1256135	Rv1130	Possible methylcitrate dehydratase PrpD
+	1256132..1257313	Rv1131	Probable methylcitrate synthase PrpC
+	1257325..1259055	Rv1132	hypothetical protein
-	1259067..1261346	Rv1133c	Probable 5-methyltetrahydropteroyltriglutamate--homocysteinemethyltransferase MetE (methionine synthase, vitamin-B12independent isozyme)

+	1261922..1262158	Rv1134	Hypothetical protein
-	1262272..1264128	Rv1135c	PPE family protein PPE16
+	1264314..1264556	Rv1135A	Possible acetyl-CoA acetyltransferase (acetoacetyl-CoA thiolase)
+	1264606..1264947	Rv1136	Possible enoyl-CoA hydratase
+	1561464..1561772	Rv1386	PE family protein PE15
+	1561769..1563388	Rv1387	PPE family protein PPE20
+	1563694..1564266	Rv1388	Putative integration host factor MihF
+	1564401..1565027	Rv1389	Probable guanylate kinase Gmk
+	1565093..1565425	Rv1390	Probable DNA-directed RNA polymerase (omega chain) RpoZ (transcriptase omega chain) (RNA polymeraseomega subunit)
+	1565441..1566697	Rv1391	Probable DNA/pantothenate metabolism flavoprotein homolog Dfp
+	1566825..1568036	Rv1392	Probable S-adenosylmethionine synthetase MetK (mat) (AdoMet synthetase) (methionineadenosyltransferase)
-	1568109..1569587	Rv1393c	Probable monoxygenase
-	1569584..1570969	Rv1394c	Probable cytochrome P450 132 Cyp132
+	1571047..1572081	Rv1395	Transcriptional regulatory protein
-	1572127..1573857	Rv1396c	PE-PGRS family protein PE_PGRS25
-	1574112..1574513	Rv1397c	Possible toxin VapC10
-	1574510..1574767	Rv1398c	Possible antitoxin VapB10
-	1574850..1575809	Rv1399c	Probable non lipolytic carboxylesterase NlhH
-	1575834..1576796	Rv1400c	Probable lipase LipH
+	1576930..1577532	Rv1401	Possible membrane protein
+	1577613..1579580	Rv1402	Putative primosomal protein N' PriA (replication factor Y)
-	1579598..1580422	Rv1403c	Putative methyltransferase
+	1580591..1581073	Rv1404	Probable transcriptional regulatory protein
-	1581145..1581969	Rv1405c	Putative methyltransferase
+	1582166..1583104	Rv1406	Probable methionyl-tRNA formyltransferase Fmt
+	1583101..1584474	Rv1407	Probable Fmu protein (sun protein)

+	1584499..1585197	Rv1408	Probable ribulose-phosphate 3-epimerase Rpe (PPE) (R5P3E) (pentose-5-phosphate 3-epimerase)
+	1585194..1586213	Rv1409	Probable bifunctional riboflavin biosynthesis protein RibG : diaminohydroxyphosphoribosylaminopyrimidinedeaminase (riboflavin-specific deaminase) +5-amino-6-(5-phosphoribosylamino) uracil reductase (HTPreductase)
-	1586210..1587766	Rv1410c	Aminoglycosides/tetracycline-transport integral membrane protein
-	1587772..1588482	Rv1411c	Conserved lipoprotein LprG
+	1588567..1589172	Rv1412	Probable riboflavin synthase alpha chain RibC (RibE)
+	1589386..1589901	Rv1413	hypothetical protein
+	1589891..1590292	Rv1414	hypothetical protein
+	1590397..1591674	Rv1415	Probable riboflavin biosynthesis protein RibA2 : GTP cyclohydrolase II + 3,4-dihydroxy-2-butanone4-phosphate synthase (DHBP synthase)
+	1591671..1592153	Rv1416	Probable riboflavin synthase beta chain RibH (6,7-dimethyl-8-ribityllumazine synthase) (DMRL synthase)(lumazine synthase)
+	1592150..1592614	Rv1417	Possible conserved membrane protein
+	1592639..1593325	Rv1418	Probable lipoprotein LprH
+	1593505..1593978	Rv1419	hypothetical protein
+	1594042..1595982	Rv1420	Probable excinuclease ABC (subunit C-nuclease) UvrC
+	1595979..1596884	Rv1421	hypothetical protein
+	1596881..1597909	Rv1422	hypothetical protein
+	1597906..1598883	Rv1423	Probable transcriptional regulatory protein WhiA
-	1598893..1599654	Rv1424c	Possible membrane protein
+	1599658..1601037	Rv1425	Possible triacylglycerol synthase (diacylglycerol acyltransferase)
-	1601059..1602321	Rv1426c	Probable esterase LipO
-	1602321..1603928	Rv1427c	Possible long-chain-fatty-acid--CoA ligase FadD12 (fatty-acid-CoA synthetase) (fatty-acid-CoAsynthase)
-	1603932..1604759	Rv1428c	hypothetical protein
+	1604878..1606146	Rv1429	hypothetical protein
+	1606386..1607972	Rv1430	PE family protein PE16
+	1608083..1609852	Rv1431	hypothetical protein
+	1609849..1611270	Rv1432	Probable dehydrogenase

+	1611434..1612249	Rv1433	Possible conserved exported protein
+	1612256..1612393	Rv1434	Hypothetical protein
-	1612342..1612950	Rv1435c	Probable conserved proline, glycine, valine-rich secreted protein
+	1613307..1614326	Rv1436	Probable glyceraldehyde 3-phosphate dehydrogenase Gap (GAPDH)
+	1614329..1615567	Rv1437	Probable phosphoglycerate kinase Pgk
+	1615564..1616349	Rv1438	Probable triosephosphate isomerase Tpi (TIM)
-	1616961..1617386	Rv1439c	hypothetical protein
+	1617837..1618070	Rv1440	Probable protein-export membrane protein (translocase subunit) SecG
-	1618209..1619684	Rv1441c	PE-PGRS family protein PE_PGRS26
+	1619791..1622091	Rv1442	Probable biotin sulfoxide reductase BisC (BDS reductase) (BSO reductase)
-	1622207..1622692	Rv1443c	hypothetical protein
-	1623287..1623697	Rv1444c	hypothetical protein
-	1623714..1624457	Rv1445c	Probable 6-phosphogluconolactonase DevB (6PGL)
-	1624454..1625365	Rv1446c	Putative OXPP cycle protein OpcA
-	1625418..1626962	Rv1447c	Probable glucose-6-phosphate 1-dehydrogenase Zwf2 (G6PD)
-	1626959..1628080	Rv1448c	Probable transaldolase Tal
-	1628097..1630199	Rv1449c	Transketolase Tkt (TK)
-	1630638..1634627	Rv1450c	PE-PGRS family protein PE_PGRS27
-	3133709..3134593	Rv2826c	Hypothetical protein
-	3134596..3135483	Rv2827c	Hypothetical protein
-	3135788..3136333	Rv2828c	hypothetical protein
-	3136330..3136599	Rv2828A	hypothetical protein
-	3136620..3137012	Rv2829c	Possible toxin VapC22
-	3137009..3137224	Rv2830c	Possible antitoxin VapB22
+	3137271..3138020	Rv2831	Probable enoyl-CoA hydratase EchA16 (enoyl hydrase) (unsaturated acyl-CoA hydratase) (crotonase)
-	3138099..3139181	Rv2832c	Probable Sn-glycerol-3-phosphate transport ATP-binding protein ABC transporter UgpC

-	3139174..3140484	Rv2833c	Probable Sn-glycerol-3-phosphate-binding lipoprotein UgpB
-	3140487..3141314	Rv2834c	Probable Sn-glycerol-3-phosphate transport integral membrane protein ABC transporter UgpE
-	3141311..3142222	Rv2835c	Probable Sn-glycerol-3-phosphate transport integral membrane protein ABC transporter UgpA
-	3142309..3143628	Rv2836c	Possible DNA-damage-inducible protein F DinF
-	3143635..3144645	Rv2837c	hypothetical protein
-	3144620..3145171	Rv2838c	Probable ribosome-binding factor a RbfA (P15B protein)
-	3145171..3147873	Rv2839c	Probable translation initiation factor if-2 InfB
-	3147959..3148258	Rv2840c	hypothetical protein
-	3148385..3149428	Rv2841c	Probable N utilization substance protein A NusA
-	3149425..3149976	Rv2842c	hypothetical protein
+	3150171..3150716	Rv2843	Probable conserved transmembrane alanine rich protein
+	3150713..3151201	Rv2844	Conserved alanine rich protein
-	3151202..3152950	Rv2845c	Probable prolyl-tRNA synthetase ProS (proline--tRNA ligase) (PRORS) (global RNA synthesisfactor) (proline translase)
-	3153039..3154631	Rv2846c	Possible integral membrane efflux protein EfpA
-	3154654..3155871	Rv2847c	Possible multifunctional enzyme siroheme synthase CysG: uroporphyrin-III C-methyltransferase(urogen III methylase) (SUMT) (uroporphyrinogen IIImethylase) (UROM) + precorrin-2 oxidase + ferrochelatase
-	3156148..3157521	Rv2848c	Probable cobyrinic acid A,C-diamide synthase CobB
-	3157521..3158144	Rv2849c	Probable cob(I)alamin adenosyltransferase CobO (corrinoid adenosyltransferase) (corrinoid adotransferaseactivity)
-	3158165..3160054	Rv2850c	Possible magnesium chelatase
-	3160051..3160521	Rv2851c	GCN5-related N-acetyltransferase
-	3160580..3162061	Rv2852c	Probable malate:quinone oxidoreductase Mqo (malate dehydrogenase [acceptor])
+	3162268..3164115	Rv2853	PE-PGRS family protein PE_PGRS48
+	3770773..3771048	Rv3357	Antitoxin RelJ
+	3771045..3771302	Rv3358	Toxin RelK
+	3771344..3772534	Rv3359	Possible oxidoreductase

+	3772651..3773019	Rv3360	hypothetical protein
-	3773016..3773567	Rv3361c	hypothetical protein
-	3773574..3774155	Rv3362c	Probable ATP/GTP-binding protein
-	3774136..3774504	Rv3363c	hypothetical protein
-	3774482..3774874	Rv3364c	hypothetical protein
-	3774871..3777501	Rv3365c	hypothetical protein
+	3777737..3778201	Rv3366	Probable tRNA/rRNA methylase SpoU (tRNA/rRNA methyltransferase)
+	3778568..3780334	Rv3367	PE-PGRS family protein PE_PGRS51
-	3780335..3780979	Rv3368c	Possible oxidoreductase
+	3780978..3781412	Rv3369	hypothetical protein
-	3781501..3784740	Rv3370c	Probable DNA polymerase III (alpha chain) DnaE2 (DNA nucleotidyltransferase)
+	3784932..3786272	Rv3371	Possible triacylglycerol synthase (diacylglycerol acyltransferase)
+	3786314..3787489	Rv3372	Trehalose 6-phosphate phosphatase OtsB2 (trehalose-phosphatase) (TPP)
+	3787726..3788367	Rv3373	Probable enoyl-CoA hydratase EchA18 (enoyl hydrase) (unsaturated acyl-CoA hydratase) (crotonase)
+	3788368..3788616	Rv3374	Probable enoyl-CoA hydratase (fragment) EchA18.1 (enoyl hydrase) (unsaturated acyl-CoA hydratase)(crotonase)
+	3788621..3790048	Rv3375	Probable amidase AmiD (acylamidase) (acylase)
+	3790156..3790809	Rv3376	hypothetical protein
-	3790848..3792353	Rv3377c	Halimadienyl diphosphate synthase
-	3792358..3793248	Rv3378c	Diterpene synthase
-	3793257..3794867	Rv3379c	Probable 1-deoxy-D-xylulose 5-phosphate synthase Dxs2 (1-deoxyxylulose-5-phosphate synthase) (DXP synthase)(DXPS)
-	3795100..>3796086	Rv3380c	Probable transposase
-	3796035..3796361	Rv3381c	Probable transposase for insertion sequence element IS6110 (fragment)
-	3796448..3797437	Rv3382c	Probable LYTB-related protein LytB1
-	3797437..3798489	Rv3383c	Possible polyprenyl synthetase IdsB (polyprenyl transferase) (polyprenyl diphosphate synthase)
-	3799243..3799635	Rv3384c	Possible toxin VapC46 Contains PIN domain
-	3799635..3799943	Rv3385c	Possible antitoxin VapB46

+	3800092..3800796	Rv3386	Possible transposase
+	3800786..3801463	Rv3387	Possible transposase
+	3801653..3803848	Rv3388	PE-PGRS family protein PE_PGRS52
-	3803919..3804791	Rv3389c	Probable 3-hydroxyacyl-thioester dehydratase HtdY
+	3804865..3805575	Rv3390	Probable conserved lipoprotein LpqD
+	3805621..3807573	Rv3391	Possible multi-functional enzyme with acyl-CoA-reductase activity AcrA1
-	3807574..3808437	Rv3392c	Cyclopropane-fatty-acyl-phospholipid synthase 1 CmaA1 (cyclopropane fatty acid synthase) (CFA synthase)(cyclopropane mycolic acid synthase 1)
