# Supplementary material for: Chromosomal rearrangements and protein globularity changes in Mycobacterium tuberculosis isolates from cerebrospinal fluid
Source: PeerJ. 2016 Sep 21;4:e2484. doi: 10.7717/peerj.2484 (PMC5036109; doi:10.7717/peerj.2484)
Supplement: Supplemental Information 10 [file peerj-04-2484-s010.pdf]

Strand	Location	Gene	Gene Description
-	332708..333136	Rv0277c	Possible toxin VapC25 Contains PIN domain
-	333160..333417	Rv0277A	Possible antitoxin VapB25
-	333437..336310	Rv0278c	PE-PGRS family protein PE_PGRS3
-	336560..339073	Rv0279c	PE-PGRS family protein PE_PGRS4
+	339364..340974	Rv0280	PPE family protein PPE3
+	340998..341906	Rv0281	Possible S-adenosylmethionine-dependent methyltransferase
+	342130..344025	Rv0282	ESX conserved component EccA3 ESX-3 type VII secretion system protein
+	344022..345638	Rv0283	ESX conserved component EccB3 ESX-3 type VII secretion system protein Possible membrane protein
+	345635..349627	Rv0284	ESX conserved component EccC3 ESX-3 type VII secretion system protein Possible membrane protein
+	349624..349932	Rv0285	PE family protein PE5
+	349935..351476	Rv0286	PPE family protein PPE4
+	351525..351818	Rv0287	ESAT-6 like protein EsxG (conserved protein TB9.8)
+	351848..352138	Rv0288	Low molecular weight protein antigen 7 EsxH (10 kDa antigen) (CFP-7) (protein TB10.4)
+	352149..353036	Rv0289	ESX-3 secretion-associated protein EspG3
+	353083..354501	Rv0290	ESX conserved component EccD3 ESX-3 type VII secretion system protein Probable transmembrane protein
+	354498..355883	Rv0291	Probable membrane-anchored mycosin MycP3 (serine protease) (subtilisin-like protease) (subtilase-like)(mycosin-3)
+	355880..356875	Rv0292	ESX conserved component EccE3 ESX-3 type VII secretion system protein Probable transmembrane protein
-	356862..358064	Rv0293c	hypothetical protein
+	358171..358956	Rv0294	Probable trans-aconitate methyltransferase Tam
-	358945..359748	Rv0295c	hypothetical protein
-	359758..361155	Rv0296c	Probable sulfatase
+	361334..363109	Rv0297	PE-PGRS family protein PE_PGRS5
+	363252..363479	Rv0298	Hypothetical protein
+	363476..363778	Rv0299	Hypothetical protein
+	363826..364047	Rv0300	Possible antitoxin VapB2

+	364044..364469	Rv0301	Possible toxin VapC2
+	364605..365237	Rv0302	Probable transcriptional regulatory protein (probably TetR/AcrR-family)
+	365234..366142	Rv0303	Probable dehydrogenase/reductase
-	366150..372764	Rv0304c	PPE family protein PPE5
-	372820..375711	Rv0305c	PPE family protein PPE6
+	375914..376585	Rv0306	Putative oxidoreductase
-	376573..377055	Rv0307c	hypothetical protein
+	377113..377829	Rv0308	Probable conserved integral membrane protein
+	377931..378587	Rv0309	Possible conserved exported protein
-	378657..379148	Rv0310c	hypothetical protein
+	379172..380401	Rv0311	hypothetical protein
+	380556..382418	Rv0312	Conserved hypothetical proline and threonine rich protein
+	382490..382876	Rv0313	hypothetical protein
-	382879..383541	Rv0314c	Possible conserved membrane protein
+	383602..384486	Rv0315	Possible beta-1,3-glucanase precursor
+	384535..385149	Rv0316	Possible muconolactone isomerase
-	385173..385943	Rv0317c	Possible glycerophosphoryl diester phosphodiesterase GlpQ2 (glycerophosphodiesterphosphodiesterase)
-	386204..386274	Rvnt04	tRNA-Gly
-	386305..387099	Rv0318c	Probable conserved integral membrane protein
+	387148..387816	Rv0319	Probable pyrrolidone-carboxylate peptidase Pcp (5-oxoprolyl-peptidase) (pyroglutamyl-peptidase I) (PGP-I)(pyrase)
+	387888..388550	Rv0320	Possible conserved exported protein
+	388582..389154	Rv0321	Probable deoxycytidine triphosphate deaminase Dcd (dCTP deaminase)
+	389260..390591	Rv0322	Probable UDP-glucose 6-dehydrogenase UdgA (UDP-GLC dehydrogenase) (UDP-GLCDH) (UDPGDH)
-	390580..391251	Rv0323c	hypothetical protein
+	391352..392032	Rv0324	Possible transcriptional regulatory protein (possibly ArsR-family)
+	392039..392263	Rv0325	Hypothetical protein

+	392273..392728	Rv0326	Hypothetical protein
-	392696..394045	Rv0327c	Possible cytochrome P450 135A1 Cyp135A1
+	394111..394713	Rv0328	Possible transcriptional regulatory protein (possibly TetR/AcrR-family)
-	394694..395320	Rv0329c	hypothetical protein
-	395347..396087	Rv0330c	Hypothetical protein
+	396201..397367	Rv0331	Possible dehydrogenase/reductase
+	397442..398227	Rv0332	hypothetical protein
+	398254..398628	Rv0333	hypothetical protein
+	398658..399524	Rv0334	Alpha-D-glucose-1-phosphate thymidylyltransferase RmlA (dTDP-glucose synthase)(dTDP-glucose pyrophosphorylase)
-	399535..400050	Rv0335c	PE family protein PE6
+	400192..401703	Rv0336	Conserved 13E12 repeat family protein
-	401873..403162	Rv0337c	Probable aspartate aminotransferase AspC (transaminase A) (ASPAT)
-	403193..405841	Rv0338c	Probable iron-sulfur-binding reductase
-	405950..408448	Rv0339c	Possible transcriptional regulatory protein
+	408634..409173	Rv0340	hypothetical protein
+	409362..410801	Rv0341	Isoniazid inductible gene protein IniB
+	410838..412760	Rv0342	Isoniazid inductible gene protein IniA
+	412757..414238	Rv0343	Isoniazid inductible gene protein IniC
-	414381..414941	Rv0344c	Probable lipoprotein LpqJ
+	415050..415460	Rv0345	hypothetical protein
-	415502..416965	Rv0346c	Possible L-asparagine permease AnsP2 (L-asparagine transport protein)
+	417304..418290	Rv0347	Probable conserved membrane protein
+	418293..418946	Rv0348	Possible transcriptional regulatory protein
+	418949..419608	Rv0349	Hypothetical protein
+	419835..421712	Rv0350	Probable chaperone protein DnaK (heat shock protein 70) (heat shock 70 kDa protein) (HSP70)
+	421709..422416	Rv0351	Probable GrpE protein (HSP-70 cofactor)

+	422452..423639	Rv0352	Probable chaperone protein DnaJ1
+	423639..424019	Rv0353	Probable heat shock protein transcriptional repressor HspR (MerR family)
-	424269..424694	Rv0354c	PPE family protein PPE7
-	424777..434679	Rv0355c	PPE family protein PPE8
-	434830..435474	Rv0356c	hypothetical protein
-	435471..436769	Rv0357c	Probable adenylosuccinate synthetase PurA (imp--aspartate ligase) (ADSS) (ampsase)
+	436860..437507	Rv0358	hypothetical protein
+	437518..438297	Rv0359	Probable conserved integral membrane protein
-	438302..438739	Rv0360c	hypothetical protein
+	438822..439649	Rv0361	Probable conserved membrane protein
+	439871..441253	Rv0362	Possible Mg2+ transport transmembrane protein MgtE
-	441265..442299	Rv0363c	Probable fructose-bisphosphate aldolase Fba
+	442395..443078	Rv0364	Possible conserved transmembrane protein
-	443067..444197	Rv0365c	hypothetical protein
-	444222..444815	Rv0366c	hypothetical protein
-	444844..445233	Rv0367c	Hypothetical protein
-	445314..446525	Rv0368c	hypothetical protein
-	446531..447046	Rv0369c	Possible membrane oxidoreductase
-	447147..448043	Rv0370c	Possible oxidoreductase
-	448040..448633	Rv0371c	hypothetical protein
-	448630..449385	Rv0372c	hypothetical protein
-	449404..451803	Rv0373c	Probable carbon monoxyde dehydrogenase (large chain)
-	451800..452279	Rv0374c	Probable carbon monoxyde dehydrogenase (small chain)
-	452294..453154	Rv0375c	Probable carbon monoxyde dehydrogenase (medium chain)
-	453230..454372	Rv0376c	hypothetical protein
+	454421..455386	Rv0377	Probable transcriptional regulatory protein (probably LysR-family)

+	455637..455858	Rv0378	Conserved hypothetical glycine rich protein
+	455977..456192	Rv0379	Possible protein transport protein SecE2
-	456268..456819	Rv0380c	Possible RNA methyltransferase (RNA methylase)
-	456915..457823	Rv0381c	Hypothetical protein
-	457841..458380	Rv0382c	Probable orotate phosphoribosyltransferase PyrE (OPRT) (oprtase)
-	458461..459315	Rv0383c	Possible conserved secreted protein
-	459456..462002	Rv0384c	Probable endopeptidase ATP binding protein (chain B) ClpB (ClpB protein) (heat shock protein F84.1)
+	462135..463307	Rv0385	Probable monooxygenase
+	463411..466668	Rv0386	Probable transcriptional regulatory protein (probably LuxR/UhpA-family)
-	466672..467406	Rv0387c	hypothetical protein
-	467459..468001	Rv0388c	PPE family protein PPE9
+	468335..469594	Rv0389	Probable phosphoribosylglycinamide formyltransferase 2 PurT (GART 2) (gar transformylase 2)(5'-phosphoribosylglycinamide transformylase 2)(formate-dependent gar transformylase)
+	469591..470013	Rv0390	hypothetical protein
+	470010..471230	Rv0391	Probable O-succinylhomoserine sulfhydrylase MetZ (OSH sulfhydrylase)
-	471227..472639	Rv0392c	Probable membrane NADH dehydrogenase NdhA
+	472781..474106	Rv0393	Conserved 13E12 repeat family protein
-	474122..474841	Rv0394c	Possible secreted protein
+	474940..475344	Rv0395	Hypothetical protein
+	475350..475742	Rv0396	Hypothetical protein
+	475816..476184	Rv0397	Conserved 13E12 repeat family protein
+	476394..476642	Rv0397A	hypothetical protein
-	476679..477320	Rv0398c	Possible secreted protein
-	477327..478556	Rv0399c	Possible conserved lipoprotein LpqK
-	478566..479753	Rv0400c	Acyl-CoA dehydrogenase FadE7
+	479789..480160	Rv0401	Probable conserved transmembrane protein
-	480355..483231	Rv0402c	Probable conserved transmembrane transport protein MmpL1

-	483228..483656	Rv0403c	Probable conserved membrane protein MmpS1
+	483977..485734	Rv0404	Fatty-acid-AMP ligase FadD30 (fatty-acid-AMP synthetase) (fatty-acid-AMP synthase)
+	485731..489939	Rv0405	Probable membrane bound polyketide synthase Pks6
-	489887..490705	Rv0406c	Beta lactamase like protein
+	490783..491793	Rv0407	F420-dependent glucose-6-phosphate dehydrogenase Fgd1
+	491786..493858	Rv0408	Probable phosphate acetyltransferase Pta (phosphotransacetylase)
+	493851..495008	Rv0409	Probable acetate kinase AckA (acetokinase)
-	495062..497314	Rv0410c	Serine/threonine-protein kinase PknG (protein kinase G) (STPK G)
-	497314..498300	Rv0411c	Probable glutamine-binding lipoprotein GlnH (GLNBP)
-	498300..499619	Rv0412c	Possible conserved membrane protein
+	499713..500366	Rv0413	Possible mutator protein MutT3 (7,8-dihydro-8-oxoguanine-triphosphatase) (8-oxo-dGTPase)(dGTP pyrophosphohydrolase)
-	500350..501018	Rv0414c	Thiamine-phosphate pyrophosphorylase ThiE (TMP pyrophosphorylase) (TMP-PPASE) (thiamine-phosphatesynthase)
+	501148..502170	Rv0415	Possible thiamine biosynthesis oxidoreductase ThiO
+	502167..502373	Rv0416	Possible protein ThiS
+	502366..503124	Rv0417	Probable thiamin biosynthesis protein ThiG (thiazole biosynthesis protein)
+	503496..504998	Rv0418	Probable lipoprotein aminopeptidase LpqL
+	505086..506582	Rv0419	Possible lipoprotein peptidase LpqM
-	506561..506971	Rv0420c	Possible transmembrane protein
-	507132..507761	Rv0421c	hypothetical protein
-	507758..508555	Rv0422c	Probable phosphomethylpyrimidine kinase ThiD (HMP-phosphate kinase) (HMP-P kinase)
-	508582..510225	Rv0423c	Probable thiamine biosynthesis protein ThiC
-	510377..510652	Rv0424c	Hypothetical protein
-	510702..515321	Rv0425c	Possible metal cation transporting P-type ATPase CtpH
-	515373..515816	Rv0426c	Possible transmembrane protein
-	516017..516892	Rv0427c	Probable exodeoxyribonuclease III protein XthA (exonuclease III) (EXO III) (AP endonuclease VI)

-	516895..517803	Rv0428c	GCN5-related N-acetyltransferase
-	517803..518396	Rv0429c	Probable polypeptide deformylase Def (PDF) (formylmethionine deformylase)
+	518733..519041	Rv0430	hypothetical protein
+	519073..519567	Rv0431	Putative tuberculin related peptide
+	519600..520322	Rv0432	Periplasmic superoxide dismutase [Cu-Zn] SodC
+	520324..521454	Rv0433	hypothetical protein
+	521514..522167	Rv0434	hypothetical protein
-	522347..524533	Rv0435c	Putative conserved ATPase
-	524530..525390	Rv0436c	Probable CDP-diacylglycerol--serine O-phosphatidyltransferase PssA (PS synthase)(phosphatidylserine synthase)
-	525387..526082	Rv0437c	Possible phosphatidylserine decarboxylase Psd (PS decarboxylase)
-	526143..527360	Rv0438c	Probable molybdopterin biosynthesis protein MoeA2
-	527379..528314	Rv0439c	Probable dehydrogenase/reductase
+	528608..530230	Rv0440	60 kDa chaperonin 2 GroEL2 (protein CPN60-2) (GroEL protein 2) (65 kDa antigen) (heat shock protein 65)(cell wall protein A) (antigen A)
-	530296..530724	Rv0441c	Hypothetical protein
-	530751..532214	Rv0442c	PPE family protein PPE10
+	532396..532911	Rv0443	hypothetical protein
-	533091..533789	Rv0444c	Anti-sigma factor RskA (regulator of sigma K)
-	533833..534396	Rv0445c	Alternative RNA polymerase sigma factor SigK
-	534445..535215	Rv0446c	Possible conserved transmembrane protein
-	535224..536507	Rv0447c	Probable cyclopropane-fatty-acyl-phospholipid synthase UfaA1 (cyclopropane fatty acid synthase) (CFAsynthase)
-	536504..537169	Rv0448c	hypothetical protein
-	537229..538548	Rv0449c	hypothetical protein
-	538588..541491	Rv0450c	Probable conserved transmembrane transport protein MmpL4
-	541488..541910	Rv0451c	Probable conserved membrane protein MmpS4
+	542142..542852	Rv0452	Possible transcriptional regulatory protein
+	543174..544730	Rv0453	PPE family protein PPE11

+	544835..545185	Rv0454	hypothetical protein
-	545375..545821	Rv0455c	hypothetical protein
-	545889..546803	Rv0456c	enoyl-CoA hydratase EchA2 (enoyl hydrase) (unsaturated acyl-CoA hydratase) (crotonase)
-	547076..547357	Rv0456A	Possible toxin MazF1
-	547344..547517	Rv0456B	Possible antitoxin MazE1
-	547586..549607	Rv0457c	Probable peptidase
+	549675..551198	Rv0458	Probable aldehyde dehydrogenase
+	551198..551689	Rv0459	hypothetical protein
+	551749..551988	Rv0460	Conserved hydrophobic protein
+	552026..552550	Rv0461	Probable transmembrane protein
+	552614..554008	Rv0462	Dihydrolipoamide dehydrogenase LpdC (lipoamide reductase (NADH)) (lipoyl dehydrogenase) (dihydrolipoyldehydrogenase) (diaphorase)
+	554016..554309	Rv0463	Probable conserved membrane protein
-	554313..554885	Rv0464c	hypothetical protein
-	554882..556306	Rv0465c	Probable transcriptional regulatory protein
+	556458..557252	Rv0466	hypothetical protein
+	557527..558813	Rv0467	Isocitrate lyase Icl (isocitrase) (isocitratase)
+	558895..559755	Rv0468	3-hydroxybutyryl-CoA dehydrogenase FadB2 (beta-hydroxybutyryl-CoA dehydrogenase) (BHBD)
+	559888..560748	Rv0469	Possible mycolic acid synthase UmaA
-	560848..561711	Rv0470c	Mycolic acid synthase PcaA (cyclopropane synthase)
-	561854..562294	Rv0470A	Hypothetical protein
-	562225..562713	Rv0471c	Hypothetical protein
-	562723..563427	Rv0472c	Probable transcriptional regulatory protein (possibly TetR-family)
+	563564..564934	Rv0473	Possible conserved transmembrane protein
+	565021..565443	Rv0474	Probable transcriptional regulatory protein
+	565797..566396	Rv0475	Iron-regulated heparin binding hemagglutinin HbhA (adhesin)
+	566508..566771	Rv0476	Possible conserved transmembrane protein

+	566776..567222	Rv0477	Possible conserved secreted protein
+	567222..567896	Rv0478	Probable deoxyribose-phosphate aldolase DeoC (phosphodeoxyriboaldolase) (deoxyriboaldolase)
-	567921..568967	Rv0479c	Probable conserved membrane protein
-	568964..569806	Rv0480c	Possible amidohydrolase
-	569988..570512	Rv0481c	Hypothetical protein
+	570539..571648	Rv0482	Probable UDP-N-acetylenolpyruvoylglucosamine reductase MurB (UDP-N-acetylmuramate dehydrogenase)
+	571710..573065	Rv0483	Probable conserved lipoprotein LprQ
-	573046..573801	Rv0484c	Probable short-chain type oxidoreductase
+	573984..575300	Rv0485	Possible transcriptional regulatory protein
+	575033..575069	RVnc0019	Fragment of putative small regulatory RNA
+	575348..576790	Rv0486	Glycosyltransferase MshA
+	576787..577338	Rv0487	hypothetical protein
+	577664..578269	Rv0488	Probable conserved integral membrane protein
+	578426..579175	Rv0489	Probable phosphoglycerate mutase 1 Gpm1 (phosphoglyceromutase) (PGAM) (BPG-dependent PGAM)
+	579349..580581	Rv0490	Putative two component sensor histidine kinase SenX3
+	580809..581492	Rv0491	Two component sensory transduction protein RegX3 (transcriptional regulatory protein) (probablyLuxR-family)
-	581489..583378	Rv0492c	Probable oxidoreductase GMC-type
-	583375..583704	Rv0492A	Hypothetical protein
-	583701..584690	Rv0493c	hypothetical protein
+	584695..585423	Rv0494	Probable transcriptional regulatory protein (probably GntR-family)
-	585424..586314	Rv0495c	hypothetical protein
+	586394..587380	Rv0496	hypothetical protein
+	587377..588309	Rv0497	Probable conserved transmembrane protein
+	588325..589167	Rv0498	hypothetical protein
+	589183..590058	Rv0499	hypothetical protein
+	590083..590970	Rv0500	Probable pyrroline-5-carboxylate reductase ProC (P5CR) (P5C reductase)

+	591111..591347	Rv0500A	hypothetical protein
+	591475..591576	Rv0500B	hypothetical protein
+	591654..592784	Rv0501	Possible UDP-glucose 4-epimerase GalE2 (galactowaldenase) (UDP-galactose 4-epimerase) (uridinediphosphate galactose 4-epimerase) (uridinediphospho-galactose 4-epimerase)
+	592791..593867	Rv0502	hypothetical protein
-	593871..594779	Rv0503c	Cyclopropane-fatty-acyl-phospholipid synthase 2 CmaA2 (cyclopropane fatty acid synthase) (CFA synthase)(cyclopropane mycolic acid synthase 2) (mycolic acidtrans-cyclopropane synthetase)
-	594802..595302	Rv0504c	hypothetical protein
-	595464..596585	Rv0505c	Possible phosphoserine phosphatase SerB1 (PSP) (O-phosphoserine phosphohydrolase) (pspase)
+	596759..597202	Rv0506	Probable conserved membrane protein MmpS2
+	597199..600105	Rv0507	Probable conserved transmembrane transport protein MmpL2
+	600098..600391	Rv0508	hypothetical protein
+	600441..601847	Rv0509	Probable glutamyl-tRNA reductase HemA (GLUTR)
+	601857..602786	Rv0510	Probable porphobilinogen deaminase HemC (PBG) (hydroxymethylbilane synthase) (HMBS)(pre-uroporphyrinogen synthase)
+	602819..604516	Rv0511	Probable uroporphyrin-III C-methyltransferase HemD (uroporphyrinogen III methylase) (urogen IIImethylase) (SUMT) (urogen III methylase) (UROM)
+	604602..605591	Rv0512	Probable delta-aminolevulinic acid dehydratase HemB (porphobilinogen synthase) (ALAD) (ALADH)
+	605604..606152	Rv0513	Possible conserved transmembrane protein
+	606149..606448	Rv0514	Possible transmembrane protein
+	606551..608062	Rv0515	Conserved 13E12 repeat family protein
-	608059..608535	Rv0516c	Possible anti-anti-sigma factor
+	608746..610056	Rv0517	Possible membrane acyltransferase
+	610188..610883	Rv0518	Possible exported protein
-	611172..612074	Rv0519c	Possible conserved membrane protein
+	612255..612605	Rv0520	Possible methyltransferase/methylase (fragment)
+	612598..612903	Rv0521	Possible methyltransferase/methylase (fragment)

+	613038..614342	Rv0522	Probable GABA permease GabP (4-amino butyrate transport carrier) (GAMA-aminobutyrate permease)
-	614326..614721	Rv0523c	hypothetical protein
+	614835..616223	Rv0524	Probable glutamate-1-semialdehyde 2,1-aminomutase HemL (GSA) (glutamate-1-semialdehydeaminotransferase) (GSA-at)
+	616223..616831	Rv0525	hypothetical protein
+	616846..617496	Rv0526	Possible thioredoxin protein (thiol-disulfide interchange protein)
+	617493..618272	Rv0527	Possible cytochrome C-type biogenesis protein CcdA
+	618305..619894	Rv0528	Probable conserved transmembrane protein
+	619891..620865	Rv0529	Possible cytochrome C-type biogenesis protein CcsA
+	620907..622124	Rv0530	hypothetical protein
-	622121..622282	Rv0530A	hypothetical protein
+	622329..622646	Rv0531	Possible conserved membrane protein
+	622793..624577	Rv0532	PE-PGRS family protein PE_PGRS6
-	624473..625480	Rv0533c	3-oxoacyl-[acyl-carrier-protein] synthase III FabH (beta-ketoacyl-ACP synthase III) (KAS III)
-	625562..626440	Rv0534c	1,4-dihydroxy-2-naphthoate octaprenyltransferase MenA (DHNA-octaprenyltransferase)
+	626457..627251	Rv0535	Probable 5'-methylthioadenosine phosphorylase Pnp (MTA phosphorylase)
+	627248..628288	Rv0536	Probable UDP-glucose 4-epimerase GalE3 (galactowaldenase) (UDP-galactose 4-epimerase) (uridinediphosphate galactose 4-epimerase) (uridinediphospho-galactose 4-epimerase)
-	628298..629731	Rv0537c	Probable integral membrane protein
+	630040..631686	Rv0538	Possible conserved membrane protein
+	631743..632375	Rv0539	Probable dolichyl-phosphate sugar synthase (dolichol-phosphate sugar synthetase) (dolichol-phosphatesugar transferase) (sugar phosphoryldolichol synthase)
+	632372..633034	Rv0540	hypothetical protein
-	633055..634404	Rv0541c	Probable conserved integral membrane protein
-	634416..635504	Rv0542c	Possible O-succinylbenzoic acid--CoA ligase MenE (OSB-CoA synthetase) (O-succinylbenzoate-CoA synthase)
-	635573..635875	Rv0543c	hypothetical protein
-	635935..636213	Rv0544c	Possible conserved transmembrane protein
-	636210..637463	Rv0545c	Probable low-affinity inorganic phosphate transporter integral membrane protein PitA

-	637583..637969	Rv0546c	hypothetical protein
-	638032..638916	Rv0547c	Possible oxidoreductase
-	639012..639956	Rv0548c	Naphthoate synthase MenB (dihydroxynaphthoic acid synthetase) (DHNA synthetase)
-	640228..640641	Rv0549c	Possible toxin VapC3
-	640638..640904	Rv0550c	Possible antitoxin VapB3
-	641096..642811	Rv0551c	Probable fatty-acid-CoA ligase FadD8 (fatty-acid-CoA synthetase) (fatty-acid-CoA synthase)
+	642889..644493	Rv0552	hypothetical protein
+	644490..645470	Rv0553	Probable muconate cycloisomerase MenC (cis,cis-muconate lactonizing enzyme) (MLE)
+	645467..646255	Rv0554	Possible peroxidase BpoC (non-haem peroxidase)
+	646298..647962	Rv0555	Probable bifunctional menaquinone biosynthesis protein MenD :2-succinyl-6-hydroxy-2,4-cyclohexadiene-1-carboxylatesynthase (SHCHC synthase) + 2-oxoglutarate decarboxylase(alpha-ketoglutarate decarboxylase) (KDC)
+	647959..648474	Rv0556	Probable conserved transmembrane protein
+	648536..649672	Rv0557	Mannosyltransferase MgtA
+	649689..650393	Rv0558	Probable ubiquinone/menaquinone biosynthesis methyltransferase MenH (2-heptaprenyl-1,4-naphthoquinonemethyltransferase)
-	650407..650745	Rv0559c	Possible conserved secreted protein
-	650779..651504	Rv0560c	Possible benzoquinone methyltransferase (methylase)
-	651529..652755	Rv0561c	Possible oxidoreductase
+	652771..653778	Rv0562	Probable polyprenyl-diphosphate synthase GrcC1 (polyprenyl pyrophosphate synthetase)
+	653879..654739	Rv0563	Probable protease transmembrane protein heat shock protein HtpX
-	654924..655949	Rv0564c	Probable glycerol-3-phosphate dehydrogenase [NAD(P)+] GpdA1 (NAD(P)H-dependent glycerol-3-phosphatedehydrogenase) (NAD(P)H-dependentdihydroxyacetone-phosphate reductase)
-	656010..657470	Rv0565c	Probable monooxygenase
-	657548..658039	Rv0566c	hypothetical protein
+	658109..658189	Rvnt05	tRNA-Tyr
+	658321..659340	Rv0567	Probable methyltransferase/methylase

+	659450..660868	Rv0568	Possible cytochrome P450 135B1 Cyp135B1
+	661003..661269	Rv0569	hypothetical protein
+	661295..663373	Rv0570	Probable ribonucleoside-diphosphate reductase (large subunit) NrdZ (ribonucleotide reductase)
-	663487..664818	Rv0571c	hypothetical protein
-	665042..665383	Rv0572c	Hypothetical protein
-	665851..667242	Rv0573c	Nicotinic acid phosphoribosyltransferase PncB2
-	667252..668394	Rv0574c	hypothetical protein
-	668579..669745	Rv0575c	Possible oxidoreductase
+	669848..671152	Rv0576	Probable transcriptional regulatory protein (possibly ArsR-family)
+	671166..671951	Rv0577	Conserved protein TB27.3
-	671996..675916	Rv0578c	PE-PGRS family protein PE_PGRS7
+	676238..676996	Rv0579	hypothetical protein
-	677125..677616	Rv0580c	hypothetical protein
+	677710..677925	Rv0581	Possible antitoxin VapB26
+	677922..678329	Rv0582	Possible toxin VapC26 Contains PIN domain
-	678389..679075	Rv0583c	Probable conserved lipoprotein LpqN
+	679229..681862	Rv0584	Possible conserved exported protein
-	681885..684272	Rv0585c	Probable conserved integral membrane protein
+	684410..685132	Rv0586	Probable transcriptional regulatory protein Mce2R (GntR-family)
+	685129..685926	Rv0587	Conserved hypothetical integral membrane protein YrbE2A
+	685928..686815	Rv0588	Conserved hypothetical integral membrane protein YrbE2B
+	686821..688035	Rv0589	Mce-family protein Mce2A
+	688032..688859	Rv0590	Mce-family protein Mce2B
+	688808..689062	Rv0590A	Mce-family related protein
+	689059..690504	Rv0591	Mce-family protein Mce2C
+	690501..692027	Rv0592	Mce-family protein Mce2D

+	692024..693232	Rv0593	Possible Mce-family lipoprotein LprL (Mce-family lipoprotein Mce2E)
+	693237..694787	Rv0594	Mce-family protein Mce2F
-	694839..695231	Rv0595c	Possible toxin VapC4
-	695228..695485	Rv0596c	Possible antitoxin VapB4
-	695668..696903	Rv0597c	hypothetical protein
-	697154..697567	Rv0598c	Possible toxin VapC27 Contains PIN domain
-	697564..697800	Rv0599c	Possible antitoxin VapB27
-	697904..698410	Rv0600c	Two component sensor kinase [second part]
-	698524..698994	Rv0601c	Two component sensor kinase [first part]
-	699038..699799	Rv0602c	Two component DNA binding transcriptional regulatory protein TcrA
+	699856..700167	Rv0603	Possible exported protein
+	700239..701189	Rv0604	Probable conserved lipoprotein LpqO
+	701406..702014	Rv0605	Possible resolvase
+	702016..702759	Rv0606	Possible transposase (fragment)
+	702813..703199	Rv0607	Hypothetical protein
+	703244..703489	Rv0608	Possible antitoxin VapB28
+	703486..703887	Rv0609	Possible toxin VapC28 Contains PIN domain
+	703830..704057	Rv0609A	hypothetical protein
+	704187..704247	RVnc0005	Putative small regulatory RNA
-	704752..705909	Rv0610c	Hypothetical protein
-	705961..706344	Rv0611c	Hypothetical protein
+	706324..706929	Rv0612	hypothetical protein
-	706948..709515	Rv0613c	hypothetical protein
+	709356..710348	Rv0614	hypothetical protein
+	710345..710587	Rv0615	Probable integral membrane protein
-	710584..710850	Rv0616c	Hypothetical protein

+	710782..711009	Rv0616A	Possible antitoxin VapB29
+	711006..711407	Rv0617	Possible toxin VapC29 Contains PIN domain
+	711536..712231	Rv0618	Probable galactose-1-phosphate uridylyltransferase GalTa [first part]
+	<712174..712719	Rv0619	Probable galactose-1-phosphate uridylyltransferase GalTb [second part]
+	712716..713807	Rv0620	Probable galactokinase GalK (galactose kinase)
+	714202..715266	Rv0621	Possible membrane protein
+	715370..716317	Rv0622	Possible membrane protein
+	716410..716664	Rv0623	Possible antitoxin VapB30
+	716664..717059	Rv0624	Possible toxin VapC30 Contains PIN domain
-	717153..717893	Rv0625c	Probable conserved transmembrane protein
+	718025..718285	Rv0626	Possible antitoxin VapB5
+	718282..718689	Rv0627	Possible toxin VapC5
-	718761..719912	Rv0628c	hypothetical protein
-	720005..721732	Rv0629c	Probable exonuclease V (alpha chain) RecD (exodeoxyribonuclease V alpha chain) (exodeoxyribonucleaseV polypeptide)
-	721729..725013	Rv0630c	Probable exonuclease V (beta chain) RecB (exodeoxyribonuclease V beta chain)(exodeoxyribonuclease Vpolypeptide) (chi-specific endonuclease)
-	725013..728306	Rv0631c	Probable exonuclease V (gamma chain) RecC (exodeoxyribonuclease V gamma chain)(exodeoxyribonucleaseV polypeptide)
-	728583..729278	Rv0632c	Probable enoyl-CoA hydratase EchA3 (enoyl hydrase) (unsaturated acyl-CoA hydratase) (crotonase)
-	729327..730166	Rv0633c	Possible exported protein
-	730320..731033	Rv0634c	Possible glyoxalase II (hydroxyacylglutathione hydrolase) (GLX II)
+	731113..731364	Rv0634A	hypothetical protein
+	731494..731566	Rvnt06	tRNA-Thr
+	731603..731676	Rvnt07	tRNA-Met
+	731712..731879	Rv0634B	50S ribosomal protein L33 RpmG2
+	731930..732406	Rv0635	(3R)-hydroxyacyl-ACP dehydratase subunit HadA
+	732393..732821	Rv0636	(3R)-hydroxyacyl-ACP dehydratase subunit HadB

+	732825..733325	Rv0637	(3R)-hydroxyacyl-ACP dehydratase subunit HadC
+	733524..733596	Rvnt08	tRNA-Trp
+	733737..734222	Rv0638	Probable preprotein translocase SecE1
+	734254..734970	Rv0639	Probable transcription antitermination protein NusG
+	735022..735450	Rv0640	50S ribosomal protein L11 RplK
+	735517..736224	Rv0641	50S ribosomal protein L1 RplA
-	736298..737203	Rv0642c	Methoxy mycolic acid synthase 4 MmaA4 (methyl mycolic acid synthase 4) (MMA4) (hydroxy mycolic acidsynthase)
-	737268..738149	Rv0643c	Methoxy mycolic acid synthase 3 MmaA3 (methyl mycolic acid synthase 3) (MMA3) (hydroxy mycolic acidsynthase)
-	738297..739160	Rv0644c	Methoxy mycolic acid synthase 2 MmaA2 (methyl mycolic acid synthase 2) (MMA2) (hydroxy mycolic acidsynthase)
-	739327..740187	Rv0645c	Methoxy mycolic acid synthase 1 MmaA1 (methyl mycolic acid synthase 1) (MMA1) (hydroxy mycolic acidsynthase)
-	740234..741139	Rv0646c	Probable lipase/esterase LipG
-	741151..742617	Rv0647c	hypothetical protein
+	742719..746366	Rv0648	Alpha-mannosidase
+	746363..747037	Rv0649	Possible malonyl CoA-acyl carrier protein transacylase FabD2 (MCT)
+	747037..747945	Rv0650	Possible sugar kinase
+	748276..748812	Rv0651	50S ribosomal protein L10 RplJ
+	748849..749241	Rv0652	50S ribosomal protein L7/L12 RplL (SA1)
-	749234..749929	Rv0653c	Possible transcriptional regulatory protein (probably TetR-family)
+	750000..751505	Rv0654	Probable dioxygenase
+	751517..752596	Rv0655	Possible ribonucleotide-transport ATP-binding protein ABC transporter Mkl
-	752984..753367	Rv0656c	Possible toxin VapC6
-	753462..753617	Rv0657c	Possible antitoxin VapB6
-	753693..754409	Rv0658c	Probable conserved integral membrane protein
-	754685..754993	Rv0659c	Toxin MazF2
-	754980..755225	Rv0660c	Possible antitoxin MazE2
-	755335..755772	Rv0661c	Possible toxin VapC7

-	755769..756023	Rv0662c	Possible antitoxin VapB7
+	756137..758500	Rv0663	Possible arylsulfatase AtsD (aryl-sulfate sulphohydrolase) (arylsulphatase)
+	758532..758804	Rv0664	Possible antitoxin VapB8
+	758801..759139	Rv0665	Possible toxin VapC8
+	759136..759309	Rv0666	Possible membrane protein
+	759807..763325	Rv0667	DNA-directed RNA polymerase (beta chain) RpoB (transcriptase beta chain) (RNA polymerase beta subunit)
+	763370..767320	Rv0668	DNA-directed RNA polymerase (beta' chain) RpoC (transcriptase beta' chain) (RNA polymerase beta'subunit)
-	767684..769597	Rv0669c	Possible hydrolase
+	769792..770550	Rv0670	Probable endonuclease IV End (endodeoxyribonuclease IV) (apurinase)
+	770582..771424	Rv0671	Possible conserved lipoprotein LpqP
+	771484..773112	Rv0672	Probable acyl-CoA dehydrogenase FadE8
+	773123..774061	Rv0673	Possible enoyl-CoA hydratase EchA4 (enoyl hydrase) (unsaturated acyl-CoA hydratase) (crotonase)
+	774064..774786	Rv0674	hypothetical protein
+	774783..775574	Rv0675	Probable enoyl-CoA hydratase EchA5 (enoyl hydrase) (unsaturated acyl-CoA hydratase) (crotonase)
-	775586..778480	Rv0676c	Probable conserved transmembrane transport protein MmpL5
-	778477..778905	Rv0677c	Possible conserved membrane protein MmpS5
+	778990..779487	Rv0678	hypothetical protein
-	779543..780040	Rv0679c	Conserved threonine rich protein
-	780042..780416	Rv0680c	Probable conserved transmembrane protein
+	780721..781311	Rv0681	Probable transcriptional regulatory protein (possibly TetR-family)
+	781560..781934	Rv0682	30S ribosomal protein S12 RpsL
+	781934..782404	Rv0683	30S ribosomal protein S7 RpsG
+	782485..784590	Rv0684	Probable elongation factor G FusA1 (EF-G)
+	784821..786011	Rv0685	Probable iron-regulated elongation factor TU Tuf (EF-TU)
+	786149..786946	Rv0686	Probable membrane protein
+	787099..787926	Rv0687	Probable short-chain type dehydrogenase/reductase

+	787940..789160	Rv0688	Putative ferredoxin reductase
-	789157..789411	Rv0689c	Hypothetical protein
-	790024..791073	Rv0690c	hypothetical protein
-	791070..791666	Rv0691c	Probable transcriptional regulatory protein
+	791658..791846	Rv0691A	Mycofactocin precursor protein
+	791831..792160	Rv0692	hypothetical protein
+	792157..793332	Rv0693	Probable coenzyme PQQ synthesis protein E PqqE (coenzyme PQQ synthesis protein III)
+	793335..794525	Rv0694	Possible L-lactate dehydrogenase (cytochrome) LldD1
+	794715..795470	Rv0695	hypothetical protein
+	795519..796931	Rv0696	Probable membrane sugar transferase
+	796933..798372	Rv0697	Probable dehydrogenase
+	798833..799444	Rv0698	hypothetical protein
+	799629..799850	Rv0699	Hypothetical protein
+	800487..800792	Rv0700	30S ribosomal protein S10 RpsJ (transcription antitermination factor NusE)
+	800809..801462	Rv0701	50S ribosomal protein L3 RplC
+	801462..802133	Rv0702	50S ribosomal protein L4 RplD
+	802133..802435	Rv0703	50S ribosomal protein L23 RplW
+	802528..803370	Rv0704	50S ribosomal protein L2 RplB
+	803411..803692	Rv0705	30S ribosomal protein S19 RpsS
+	803689..804282	Rv0706	50S ribosomal protein L22 RplV
+	804282..805106	Rv0707	30S ribosomal protein S3 RpsC
+	805110..805526	Rv0708	50S ribosomal protein L16 RplP
+	805526..805759	Rv0709	50S ribosomal protein L29 RpmC
+	805756..806166	Rv0710	30S ribosomal protein S17 RpsQ
+	806335..808698	Rv0711	Possible arylsulfatase AtsA (aryl-sulfate sulphohydrolase) (arylsulphatase)
+	808746..809645	Rv0712	hypothetical protein

+	809946..810887	Rv0713	Probable conserved transmembrane protein
+	811373..811741	Rv0714	50S ribosomal protein L14 RplN
+	811742..812059	Rv0715	50S ribosomal protein L24 RplX
+	812059..812622	Rv0716	50S ribosomal protein L5 RplE
+	812627..812812	Rv0717	30S ribosomal protein S14 RpsN1
+	812976..813374	Rv0718	30S ribosomal protein S8 RpsH
+	813398..813937	Rv0719	50S ribosomal protein L6 RplF
+	813940..814308	Rv0720	50S ribosomal protein L18 RplR
+	814328..814990	Rv0721	30S ribosomal protein S5 RpsE
+	814993..815190	Rv0722	50S ribosomal protein L30 RpmD
+	815190..815630	Rv0723	50S ribosomal protein L15 RplO
+	815663..817534	Rv0724	Possible protease IV SppA (endopeptidase IV) (signal peptide peptidase)
-	817531..>817866	Rv0724A	hypothetical protein
-	817539..818444	Rv0725c	hypothetical protein
-	818537..819640	Rv0726c	Possible S-adenosylmethionine-dependent methyltransferase
-	819843..820499	Rv0727c	Possible L-fuculose phosphate aldolase FucA (L-fuculose-1-phosphate aldolase)
-	820496..821476	Rv0728c	Possible D-3-phosphoglycerate dehydrogenase SerA2 (phosphoglycerate dehydrogenase) (PGDH)
+	821507..822853	Rv0729	Possible D-xylulose kinase XylB (xylulokinase) (xylulose kinase)
+	822866..823594	Rv0730	GCN5-related N-acetyltransferase
-	823683..824639	Rv0731c	Possible S-adenosylmethionine-dependent methyltransferase
+	824800..826125	Rv0732	Probable preprotein translocase SecY
+	826122..826667	Rv0733	Adenylate kinase Adk (ATP-AMP transphosphorylase)
+	826670..827470	Rv0734	Methionine aminopeptidase MapA (map) (peptidase M) (MetAP)
+	827543..828076	Rv0735	Probable alternative RNA polymerase sigma factor SigL
+	828140..828892	Rv0736	Anti-sigma factor RslA
+	829207..829704	Rv0737	Possible transcriptional regulatory protein

+	830062..830610	Rv0738	hypothetical protein
+	830855..831661	Rv0739	hypothetical protein
+	831776..832303	Rv0740	hypothetical protein
+	832534..832848	Rv0741	Probable transposase (fragment)
+	832981..833508	Rv0742	PE-PGRS family protein PE_PGRS8
-	833886..834443	Rv0743c	Hypothetical protein
-	834440..834946	Rv0744c	Possible transcriptional regulatory protein
+	835154..835681	Rv0745	hypothetical protein
+	835701..838052	Rv0746	PE-PGRS family protein PE_PGRS9
+	838451..840856	Rv0747	PE-PGRS family protein PE_PGRS10
+	840947..841204	Rv0748	Possible antitoxin VapB31
+	841228..841656	Rv0749	Possible toxin VapC31 Contains PIN domain
+	1855764..1856696	Rv1646	PE family protein PE17
+	1856774..1857724	Rv1647	Adenylate cyclase (ATP pyrophosphate-lyase) (adenylyl cyclase)
+	1857731..1858537	Rv1648	Probable transmembrane protein
+	1858733..1859758	Rv1649	Probable phenylalanyl-tRNA synthetase, alpha chain PheS
+	1859758..1862253	Rv1650	Probable phenylalanyl-tRNA synthetase, beta chain PheT
-	1862347..1865382	Rv1651c	PE-PGRS family protein PE_PGRS30
+	1865576..1866634	Rv1652	Probable N-acetyl-gamma-glutamyl-phoshate reductase ArgC
+	1866631..1867845	Rv1653	Probable glutamate N-acetyltransferase ArgJ
+	1867842..1868726	Rv1654	Probable acetylglutamate kinase ArgB
+	1868723..1869925	Rv1655	Probable acetylornithine aminotransferase ArgD
+	1869922..1870845	Rv1656	Probable ornithine carbamoyltransferase, anabolic ArgF
+	1870842..1871354	Rv1657	Probable arginine repressor ArgR (AHRC)
+	1871363..1872559	Rv1658	Probable argininosuccinate synthase ArgG
+	1872639..1874051	Rv1659	Probable argininosuccinate lyase ArgH

+	1874160..1875221	Rv1660	Chalcone synthase Pks10
+	1875304..1881684	Rv1661	Probable polyketide synthase Pks7
+	1881704..1886512	Rv1662	Probable polyketide synthase Pks8
+	1886512..1888020	Rv1663	Probable polyketide synthase Pks17
+	1888026..1891079	Rv1664	Probable polyketide synthase Pks9
+	1891226..1892287	Rv1665	Chalcone synthase Pks11
-	1892270..1893562	Rv1666c	Probable cytochrome P450 139 Cyp139
-	1893577..1894230	Rv1667c	Probable second part of macrolide-transport ATP-binding protein ABC transporter
-	1894224..1895342	Rv1668c	Probable first part of macrolide-transport ATP-binding protein ABC transporter
+	1895725..1896087	Rv1669	Hypothetical protein
+	1896120..1896467	Rv1670	hypothetical protein
+	1896475..1896867	Rv1671	Probable membrane protein
