# Supplementary material for: Chromosomal rearrangements and protein globularity changes in Mycobacterium tuberculosis isolates from cerebrospinal fluid
Source: PeerJ. 2016 Sep 21;4:e2484. doi: 10.7717/peerj.2484 (PMC5036109; doi:10.7717/peerj.2484)
Supplement: Supplemental Information 11 [file peerj-04-2484-s011.pdf]

	Strand	Location	Gene	Gene Description
UM-CSF06	-	3839691..3840197	Rv3422c	tRNA threonylcarbamoyladenosine biosynthesis protein
	-	3840194..3841420	Rv3423c	alanine racemase
	-	3841714..3842076	Rv3424c	hypothetical protein
	+	3842239..3842769	Rv3425	PPE family protein PPE57
	+	3843036..3843734	Rv3426	PPE family protein PPE58
	-	3843885..3844640	Rv3427c	transposase
	-	3844738..3845970	Rv3428c	transposase
	+	3847165..3847701	Rv3429	PPE family protein PPE59
	-	3847642..3848805	Rv3430c	transposase
	-	3848844..3848999	Rv3430a	hypothetical protein
	-	3850372..3851754	Rv3432c	glutamate decarboxylase GadB
UM-CSF09	-	1266485..1266985	Rv1139c	hypothetical protein
	+	1267347..1268195	Rv1140	integral membrane protein
	-	1268203..1269009	Rv1141c	enoyl-CoA hydratase EchA11
	-	1269152..1269958	Rv1142c	enoyl-CoA hydratase EchA10
	+	1270062..1271144	Rv1143	alpha-methylacyl-CoA racemase
	+	1271156..1271908	Rv1144	oxidoreductase
	+	1272423..1273334	Rv1145	transmembrane transport protein
	+	1273355..1274767	Rv1146	transmembrane transport protein
UM-CSF15	Strand	Location	Gene	Gene Description
	+	1777859..1778539	Rv1570	ATP-dependent dethiobiotin synthetase BioD
	+	1778539..1779048	Rv1571	hypothetical protein
	+	1779314..1779724	Rv1573	phage protein
	+	1779930..1780241	Rv1574	phage protein
	+	1780199..1780699	Rv1575	phage protein
	-	1780643..1782064	Rv1576c	phage capsid protein
	-	1782072..1782584	Rv1577c	phage prohead protease
	-	1782758..1783228	Rv1578c	phage protein
	-	1783309..1783623	Rv1579c	phage protein
	-	1783620..1783892	Rv1580c	phage protein
	-	1783906..1784301	Rv1581c	phage protein
	-	1784497..1785912	Rv1582c	phage protein
	-	1785912..1786310	Rv1583c	phage protein
	-	1786307..1786528	Rv1584c	phage protein
	-	1786584..1787099	Rv1585c	phage protein
	-	1787096..1788505	Rv1586c	phage integrase
	-	1788162..1789163	Rv1587c	hypothetical protein
	-	1789168..1789836	Rv1588c	hypothetical protein
	+	1790284..1791333	Rv1589	biotin synthetase
	+	1791334..1791573	Rv1590	hypothetical protein
	+	1791570..1792235	Rv1591	transmembrane protein

	Strand	Location	Gene	Gene Description
UM-CSF17	+	3939617..3941761	Rv3511	PE-PGRS family protein PE_PGRS55
	+	3943812..3944963	Rv3512	PE-PGRS family protein PE_PGRS56
	-	3945092..3945748	Rv3513c	fatty-acid--CoA ligase FadD18
	+	3945794..3950263	Rv3514	PE-PGRS family protein PE_PGRS57
	-	3950824..3952470	Rv3515c	long-chain-fatty-acid--CoA ligase FadD19
	+	3952544..3953335	Rv3516	enoyl-CoA hydratase EchA19
