# Supplementary material for: Chromosomal rearrangements and protein globularity changes in Mycobacterium tuberculosis isolates from cerebrospinal fluid
Source: PeerJ. 2016 Sep 21;4:e2484. doi: 10.7717/peerj.2484 (PMC5036109; doi:10.7717/peerj.2484)
Supplement: Supplemental Information 12 [file peerj-04-2484-s012.pdf]

Gene	Rv No.	Position	Affected genes
embR	Rv1267c	372	Cys changed to Gly
embR	Rv1267c	376	Phe changed to Leu
lppD	Rv1899c	between 50 and 51	Insertion - Ala,Ser,Thr,Ala,Arg,Pro,Ala,Ala,Thr,Ala,Leu,Pro,Ala,Val,Ala
PE_PGRS10	Rv0747	447	Ala changed to Asp
PE_PGRS10	Rv0747	450	Ala changed to Asp
PE_PGRS10	Rv0747	277	Asp changed to Asn
PE_PGRS10	Rv0747	362	Ile changed to Leu
PE_PGRS10	Rv0747	457	Leu changed to Phe
PE_PGRS10	Rv0747	287	Ser changed to Ala
PE_PGRS19	Rv1067c	271	Ala changed to Val
PE_PGRS21	Rv1087	between 711 and 712	Insertion - Ala,Gly,Gly,Gly,Gly,Gly,Ala,Gly,Gly,Ile,Gly,Gly,Asp,Gly,Gly
PE_PGRS49	Rv3344c	from 164 to 173	Del - Ala,Thr,Asn,Pro,Gly,Ser,Gly,Ser,Arg,Gly
PPE58	Rv3426	107	Thr changed to Ala
PPE58	Rv3426	from 217 to 218	Del - Met,Val
Rv0278c	Rv0278c	346	Ala changed to Ser
Rv0278c	Rv0278c	842	Ala changed to Gly
Rv0278c	Rv0278c	344	Met changed to Thr
Rv0278c	Rv0278c	841	Ser changed to Tyr
