# Supplementary material for: Chromosomal rearrangements and protein globularity changes in Mycobacterium tuberculosis isolates from cerebrospinal fluid
Source: PeerJ. 2016 Sep 21;4:e2484. doi: 10.7717/peerj.2484 (PMC5036109; doi:10.7717/peerj.2484)
Supplement: Supplemental Information 13 [file peerj-04-2484-s013.pdf]

Category	Term	PValue
COG_ONTOLOGY	Transcription	3.66E-04
GOTERM_BP_FAT	GO:0006355~regulation of transcription, DNA-dependent	7.73E-07
GOTERM_BP_FAT	GO:0051252~regulation of RNA metabolic process	8.90E-07
GOTERM_BP_FAT	GO:0045449~regulation of transcription	4.39E-06
GOTERM_BP_FAT	GO:0006350~transcription	2.81E-05
GOTERM_BP_FAT	GO:0009187~cyclic nucleotide metabolic process	0.009860284
GOTERM_BP_FAT	GO:0009190~cyclic nucleotide biosynthetic process	0.009860284
GOTERM_BP_FAT	GO:0007242~intracellular signaling cascade	0.028798523
GOTERM_CC_FAT	GO:0016021~integral to membrane	4.42E-06
GOTERM_CC_FAT	GO:0031224~intrinsic to membrane	6.36E-05
GOTERM_CC_FAT	GO:0005886~plasma membrane	0.032338255
GOTERM_MF_FAT	GO:0003700~transcription factor activity	7.77E-07
GOTERM_MF_FAT	GO:0030528~transcription regulator activity	4.63E-06
GOTERM_MF_FAT	GO:0003677~DNA binding	5.14E-05
GOTERM_MF_FAT	GO:0016849~phosphorus-oxygen lyase activity	0.009764838
INTERPRO	IPR001647:Transcriptional regulator, TetR-like, DNA-binding, bacterial/archaeal	1.41E-05
INTERPRO	IPR012287:Homeodomain-related	1.59E-04
INTERPRO	IPR001279:Beta-lactamase-like	0.001356161
INTERPRO	IPR013216:Methyltransferase type 11	0.009800718
INTERPRO	IPR002641:Patatin	0.032394203
INTERPRO	IPR001054:Adenylyl cyclase class-3/4/guanylyl cyclase	0.045571002
KEGG_PATHWAY	mtu00350:Tyrosine metabolism	3.55E-10
KEGG_PATHWAY	mtu00150:Androgen and estrogen metabolism	2.19E-07
KEGG_PATHWAY	mtu00450:Selenoamino acid metabolism	1.70E-06
KEGG_PATHWAY	mtu00340:Histidine metabolism	2.48E-06
KEGG_PATHWAY	mtu00626:Naphthalene and anthracene degradation	1.84E-05
KEGG_PATHWAY	mtu00642:Ethylbenzene degradation	6.92E-04
KEGG_PATHWAY	mtu00360:Phenylalanine metabolism	0.00369836
KEGG_PATHWAY	mtu00860:Porphyrin and chlorophyll metabolism	0.014895679
KEGG_PATHWAY	mtu00780:Biotin metabolism	0.019713574
KEGG_PATHWAY	mtu03430:Mismatch repair	0.044554197
KEGG_PATHWAY	mtu00310:Lysine degradation	0.044572173
SWISS-PROT	transcription regulation	0.008901379
SWISS-PROT	dna-binding	0.009996586
SWISS-PROT	Transcription	0.010891026
