# Supplementary material for: Chromosomal rearrangements and protein globularity changes in Mycobacterium tuberculosis isolates from cerebrospinal fluid
Source: PeerJ. 2016 Sep 21;4:e2484. doi: 10.7717/peerj.2484 (PMC5036109; doi:10.7717/peerj.2484)
Supplement: Supplemental Information 14 [file peerj-04-2484-s014.pdf]

Locus	Reference
Rv0311	Be et al., 2008
Rv0805	Be et al., 2008
Rv0931c	Be et al., 2008;Be et al., 2012
Rv0986	Be et al., 2008;Jain et al., 2006
Rv0368c	Jain et al., 2006
Rv0573c	Jain et al., 2006
Rv0619	Jain et al., 2006
Rv0661c	Jain et al., 2006
Rv0662c	Jain et al., 2006
Rv0966c	Jain et al., 2006
Rv0967	Jain et al., 2006
Rv0968	Jain et al., 2006
Rv0970	Jain et al., 2006
Rv0971c	Jain et al., 2006
Rv0974c	Jain et al., 2006
Rv0975c	Jain et al., 2006
Rv0977	Jain et al., 2006
Rv0978c	Jain et al., 2006
Rv0980c	Jain et al., 2006
Rv0982	Jain et al., 2006
Rv0983	Jain et al., 2006
Rv0984	Jain et al., 2006
Rv0987	Jain et al., 2006
Rv0989c	Jain et al., 2006
Rv0990c	Jain et al., 2006
Rv0991c	Jain et al., 2006
Rv1726	Jain et al., 2006
Rv1801	Jain et al., 2006
Rv1966	Jain et al., 2006
Rv1968	Jain et al., 2006
Rv2318	Jain et al., 2006
Rv3021c	Jain et al., 2006
Rv3349c	Jain et al., 2006
Rv3351c	Jain et al., 2006
Rv3639c	Jain et al., 2006
Rv3833	Jain et al., 2006
Rv0079	Be et al., 2012
Rv0336	Be et al., 2012
Rv0727c	Be et al., 2012
Rv0755c	Be et al., 2012
Rv1273c	Be et al., 2012

Rv1673c	Be et al., 2012
Rv1914c	Be et al., 2012
Rv1932	Be et al., 2012
Rv2387	Be et al., 2012
Rv3094c	Be et al., 2012
Rv3159c	Be et al., 2012
Rv3224	Be et al., 2012
Rv3353c	Be et al., 2012
Rv1837c	Haldar et al., 2012
Rv0475	Pethe et al., 2001
Rv2947c	Tsenova et al., 2005
Rv2946c	Tsenova et al., 2005
Rv0015c	Av-Gay & Everett, 2000
Rv0014c	Av-Gay & Everett, 2000
Rv1743	Av-Gay & Everett, 2000
Rv1746	Av-Gay & Everett, 2000
Rv0410c	Av-Gay & Everett, 2000
Rv1266c	Av-Gay & Everett, 2000
Rv2914c	Av-Gay & Everett, 2000
Rv2088	Av-Gay & Everett, 2000
Rv3080c	Av-Gay & Everett, 2000
Rv2176	Av-Gay & Everett, 2000
