# Supplementary material for: Chromosomal rearrangements and protein globularity changes in Mycobacterium tuberculosis isolates from cerebrospinal fluid
Source: PeerJ. 2016 Sep 21;4:e2484. doi: 10.7717/peerj.2484 (PMC5036109; doi:10.7717/peerj.2484)
Supplement: Supplemental Information 15 [file peerj-04-2484-s015.pdf]

| Locus | Reference |
|---|---|
| SP_0019 | Molzen et. al., 2011 |
| SP_0029 | Molzen et. al. , 2011 |
| SP_0042 | Molzen et. al. , 2011 |
| SP_0058 | Molzen et. al. , 2011 |
| SP_0067 | Molzen et. al. , 2011 |
| SP_0079 | Molzen et. al. , 2011 |
| SP_0098 | Molzen et. al. , 2011 |
| SP_0099 | Molzen et. al. , 2011 |
| SP_0101 | Molzen et. al. , 2011 |
| SP_0149 | Molzen et. al. , 2011 |
| SP_0151 | Molzen et. al. , 2011 |
| SP_0152 | Molzen et. al. , 2011 |
| SP_0197 | Molzen et. al. , 2011 |
| SP_0198 | Molzen et. al. , 2011 |
| SP_0199 | Molzen et. al. , 2011 |
| SP_0276 | Molzen et. al. , 2011 |
| SP_0279 | Molzen et. al. , 2011 |
| SP_0282 | Molzen et. al. , 2011 |
| SP_0308 | Molzen et. al. , 2011 |
| SP_0342 | Molzen et. al. , 2011 |
| SP_0348 | Molzen et. al. , 2011 |
| SP_0349 | Molzen et. al. , 2011 |
| SP_0350 | Molzen et. al. , 2011 |
| SP_0351 | Molzen et. al. , 2011 |
| SP_0358 | Molzen et. al. , 2011 |
| SP_0416 | Molzen et. al. , 2011 |
| SP_0494 | Molzen et. al. , 2011 |
| SP_0552 | Molzen et. al. , 2011 |
| SP_0585 | Molzen et. al. , 2011 |
| SP_0665 | Molzen et. al. , 2011 |
| SP_0695 | Molzen et. al. , 2011 |
| SP_0731 | Molzen et. al. , 2011 |
| SP_0746 | Molzen et. al. , 2011 |
| SP_0748 | Molzen et. al. , 2011 |
| SP_0749 | Molzen et. al. , 2011 |
| SP_0751 | Molzen et. al. , 2011 |
| SP_0752 | Molzen et. al. , 2011 |
| SP_0753 | Molzen et. al. , 2011 |
| SP_0822 | Molzen et. al. , 2011 |
| SP_0823 | Molzen et. al. , 2011 |
| SP_0826 | Molzen et. al. , 2011 |
| SP_0881 | Molzen et. al. , 2011 |
| SP_0925 | Molzen et. al. , 2011 |
| SP_0931 | Molzen et. al. , 2011 |
| SP_1025 | Molzen et. al. , 2011 |
| SP_1059 | Molzen et. al. , 2011 |
| SP_1062 | Molzen et. al. , 2011 |
| SP_1063 | Molzen et. al. , 2011 |
| SP_1068 | Molzen et. al. , 2011 |

| | |
|---|---|
| SP_1069 | Molzen et. al. , 2011 |
| SP_1121 | Molzen et. al. , 2011 |
| SP_1296 | Molzen et. al. , 2011 |
| SP_1297 | Molzen et. al. , 2011 |
| SP_1298 | Molzen et. al. , 2011 |
| SP_1299 | Molzen et. al. , 2011 |
| SP_1330 | Molzen et. al. , 2011 |
| SP_1331 | Molzen et. al. , 2011 |
| SP_1336 | Molzen et. al. , 2011 |
| SP_1356 | Molzen et. al. , 2011 |
| SP_1376 | Molzen et. al. , 2011 |
| SP_1393 | Molzen et. al. , 2011 |
| SP_1462 | Molzen et. al. , 2011 |
| SP_1465 | Molzen et. al. , 2011 |
| SP_1466 | Molzen et. al. , 2011 |
| SP_1502 | Molzen et. al. , 2011 |
| SP_1507 | Molzen et. al. , 2011 |
| SP_1544 | Molzen et. al. , 2011 |
| SP_1563 | Molzen et. al. , 2011 |
| SP_1635 | Molzen et. al. , 2011 |
| SP_1645 | Molzen et. al. , 2011 |
| SP_1799 | Molzen et. al. , 2011 |
| SP_1931 | Molzen et. al. , 2011 |
| SP_1966 | Molzen et. al. , 2011 |
| SP_1995 | Molzen et. al. , 2011 |
| SP_2021 | Molzen et. al. , 2011 |
| SP_2098 | Molzen et. al. , 2011 |
| SP_2116 | Molzen et. al. , 2011 |
| SP_2198 | Molzen et. al. , 2011 |
| SP_2205 | Molzen et. al. , 2011 |
| SP_2206 | Molzen et. al. , 2011 |
| SP_2231 | Molzen et. al. , 2011 |
| SP_0088 | Orihuela et al ., 2004 |
| SP_0111 | Orihuela et al., 2004 |
| SP_0117 | Orihuela et al., 2004 |
| SP_0149 | Orihuela et al., 2004 |
| SP_0151 | Orihuela et al., 2004 |
| SP_0173 | Orihuela et al., 2004 |
| SP_0205 | Orihuela et al., 2004 |
| SP_0265 | Orihuela et al., 2004 |
| SP_0266 | Orihuela et al., 2004 |
| SP_0348 | Orihuela et al., 2004 |
| SP_0446 | Orihuela et al., 2004 |
| SP_0481 | Orihuela et al., 2004 |
| SP_0483 | Orihuela et al., 2004 |
| SP_0629 | Orihuela et al., 2004 |
| SP_0641 | Orihuela et al., 2004 |
| SP_0736 | Orihuela et al., 2004 |
| SP_0750 | Orihuela et al., 2004 |
| SP_0751 | Orihuela et al., 2004 |

| | |
|---|---|
| SP_0752 | Orihuela et al., 2004 |
| SP_0753 | Orihuela et al., 2004 |
| SP_0792 | Orihuela et al., 2004 |
| SP_0794 | Orihuela et al., 2004 |
| SP_0800 | Orihuela et al., 2004 |
| SP_0841 | Orihuela et al., 2004 |
| SP_1014 | Orihuela et al., 2004 |
| SP_1122 | Orihuela et al., 2004 |
| SP_1160 | Orihuela et al., 2004 |
| SP_1249 | Orihuela et al., 2004 |
| SP_1267 | Orihuela et al., 2004 |
| SP_1268 | Orihuela et al., 2004 |
| SP_1269 | Orihuela et al., 2004 |
| SP_1270 | Orihuela et al., 2004 |
| SP_1271 | Orihuela et al., 2004 |
| SP_1276 | Orihuela et al., 2004 |
| SP_1277 | Orihuela et al., 2004 |
| SP_1310 | Orihuela et al., 2004 |
| SP_1468 | Orihuela et al., 2004 |
| SP_1550 | Orihuela et al., 2004 |
| SP_1551 | Orihuela et al., 2004 |
| SP_1587 | Orihuela et al., 2004 |
| SP_1648 | Orihuela et al., 2004 |
| SP_1649 | Orihuela et al., 2004 |
| SP_1650 | Orihuela et al., 2004 |
| SP_1675 | Orihuela et al., 2004 |
| SP_1710 | Orihuela et al., 2004 |
| SP_1725 | Orihuela et al., 2004 |
| SP_1735 | Orihuela et al., 2004 |
| SP_1804 | Orihuela et al., 2004 |
| SP_1805 | Orihuela et al., 2004 |
| SP_1888 | Orihuela et al., 2004 |
| SP_1889 | Orihuela et al., 2004 |
| SP_1890 | Orihuela et al., 2004 |
| SP_1909 | Orihuela et al., 2004 |
| SP_1968 | Orihuela et al., 2004 |
| SP_2021 | Orihuela et al., 2004 |
| SP_2095 | Orihuela et al., 2004 |
| SP_2101 | Orihuela et al., 2004 |
| SP_2196 | Orihuela et al., 2004 |
| SP_2201 | Orihuela et al., 2004 |
| SP_2185 | Mahdi et al., 2012 |
