# Supplementary material for: Chromosomal rearrangements and protein globularity changes in Mycobacterium tuberculosis isolates from cerebrospinal fluid
Source: PeerJ. 2016 Sep 21;4:e2484. doi: 10.7717/peerj.2484 (PMC5036109; doi:10.7717/peerj.2484)
Supplement: Supplemental Information 16 [file peerj-04-2484-s016.pdf]

ID	Reference
NMA0692	Hao et al.,2011
NMA0693	Hao et al.,2011
NMA0694	Hao et al.,2011
NMA1792	Hao et al.,2011
NMA1793	Hao et al.,2011
NMA1794	Hao et al.,2011
NMA1795	Hao et al.,2011
NMA1796	Hao et al.,2011
NMA1797	Hao et al.,2011
NMA1798	Hao et al.,2011
NMA1799	Hao et al.,2011
NMB0014	Hao et al.,2011
NMB0017	Hao et al.,2011
NMB0018	Hao et al.,2011
NMB0033	Hao et al.,2011
NMB0038	Hao et al.,2011
NMB0051	Hao et al.,2011
NMB0064	Hao et al.,2011
NMB0065	Hao et al.,2011
NMB0071	Hao et al.,2011
NMB0072	Hao et al.,2011
NMB0073	Hao et al.,2011
NMB0074	Hao et al.,2011
NMB0082	Hao et al.,2011
NMB0083	Hao et al.,2011
NMB0178	Hao et al.,2011
NMB0180	Hao et al.,2011
NMB0182	Hao et al.,2011
NMB0199	Hao et al.,2011
NMB0205	Hao et al.,2011
NMB0216	Hao et al.,2011
NMB0218	Hao et al.,2011
NMB0278	Hao et al.,2011
NMB0293	Hao et al.,2011
NMB0294	Hao et al.,2011
NMB0299	Hao et al.,2011
NMB0318	Hao et al.,2011
NMB0319	Hao et al.,2011
NMB0329	Hao et al.,2011
NMB0330	Hao et al.,2011
NMB0332	Hao et al.,2011
NMB0333	Hao et al.,2011
NMB0364	Hao et al.,2011

NMB0365	Hao et al.,2011
NMB0374	Hao et al.,2011
NMB0375	Hao et al.,2011
NMB0382	Hao et al.,2011
NMB0391	Hao et al.,2011
NMB0394	Hao et al.,2011
NMB0407	Hao et al.,2011
NMB0413	Hao et al.,2011
NMB0460	Hao et al.,2011
NMB0467	Hao et al.,2011
NMB0483	Hao et al.,2011
NMB0493	Hao et al.,2011
NMB0496	Hao et al.,2011
NMB0497	Hao et al.,2011
NMB0546	Hao et al.,2011
NMB0555	Hao et al.,2011
NMB0567	Hao et al.,2011
NMB0584	Hao et al.,2011
NMB0585	Hao et al.,2011
NMB0586	Hao et al.,2011
NMB0632	Hao et al.,2011
NMB0633	Hao et al.,2011
NMB0634	Hao et al.,2011
NMB0652	Hao et al.,2011
NMB0653	Hao et al.,2011
NMB0663	Hao et al.,2011
NMB0675	Hao et al.,2011
NMB0686	Hao et al.,2011
NMB0718	Hao et al.,2011
NMB0750	Hao et al.,2011
NMB0757	Hao et al.,2011
NMB0828	Hao et al.,2011
NMB0879	Hao et al.,2011
NMB0884	Hao et al.,2011
NMB0886	Hao et al.,2011
NMB0888	Hao et al.,2011
NMB0902	Hao et al.,2011
NMB0905	Hao et al.,2011
NMB0906	Hao et al.,2011
NMB0992	Hao et al.,2011
NMB0995	Hao et al.,2011
NMB1048	Hao et al.,2011
NMB1072	Hao et al.,2011
NMB1077	Hao et al.,2011
NMB1162	Hao et al.,2011

NMB1206	Hao et al.,2011
NMB1207	Hao et al.,2011
NMB1210	Hao et al.,2011
NMB1214	Hao et al.,2011
NMB1220	Hao et al.,2011
NMB1283	Hao et al.,2011
NMB1332	Hao et al.,2011
NMB1368	Hao et al.,2011
NMB1372	Hao et al.,2011
NMB1398	Hao et al.,2011
NMB1403	Hao et al.,2011
NMB1405	Hao et al.,2011
NMB1409	Hao et al.,2011
NMB1412	Hao et al.,2011
NMB1414	Hao et al.,2011
NMB1415	Hao et al.,2011
NMB1428	Hao et al.,2011
NMB1429	Hao et al.,2011
NMB1442	Hao et al.,2011
NMB1475	Hao et al.,2011
NMB1494	Hao et al.,2011
NMB1527	Hao et al.,2011
NMB1540	Hao et al.,2011
NMB1541	Hao et al.,2011
NMB1572	Hao et al.,2011
NMB1591	Hao et al.,2011
NMB1621	Hao et al.,2011
NMB1622	Hao et al.,2011
NMB1646	Hao et al.,2011
NMB1668	Hao et al.,2011
NMB1688	Hao et al.,2011
NMB1704	Hao et al.,2011
NMB1705	Hao et al.,2011
NMB1714	Hao et al.,2011
NMB1715	Hao et al.,2011
NMB1716	Hao et al.,2011
NMB1717	Hao et al.,2011
NMB1719	Hao et al.,2011
NMB1730	Hao et al.,2011
NMB1738	Hao et al.,2011
NMB1739	Hao et al.,2011
NMB1753	Hao et al.,2011
NMB1763	Hao et al.,2011
NMB1768	Hao et al.,2011
NMB1780	Hao et al.,2011

NMB1802	Hao et al.,2011
NMB1808	Hao et al.,2011
NMB1809	Hao et al.,2011
NMB1810	Hao et al.,2011
NMB1811	Hao et al.,2011
NMB1812	Hao et al.,2011
NMB1814	Hao et al.,2011
NMB1820	Hao et al.,2011
NMB1821	Hao et al.,2011
NMB1822	Hao et al.,2011
NMB1829	Hao et al.,2011
NMB1845	Hao et al.,2011
NMB1870	Hao et al.,2011
NMB1882	Hao et al.,2011
NMB1906	Hao et al.,2011
NMB1926	Hao et al.,2011
NMB1928	Hao et al.,2011
NMB1929	Hao et al.,2011
NMB1946	Hao et al.,2011
NMB1989	Hao et al.,2011
NMB1990	Hao et al.,2011
NMB1991	Hao et al.,2011
NMB2001	Hao et al.,2011
NMB2015	Hao et al.,2011
NMB2039	Hao et al.,2011
NMB2048	Hao et al.,2011
NMB2127	Hao et al.,2011
NMB2132	Hao et al.,2011
NMB2152	Hao et al.,2011
NMB2156	Hao et al.,2011
NMB2160	Hao et al.,2011
