# Supplementary material for: Chromosomal rearrangements and protein globularity changes in Mycobacterium tuberculosis isolates from cerebrospinal fluid
Source: PeerJ. 2016 Sep 21;4:e2484. doi: 10.7717/peerj.2484 (PMC5036109; doi:10.7717/peerj.2484)
Supplement: Supplemental Information 17 [file peerj-04-2484-s017.pdf]

**Gene**	**Reference**
FimH	Pouttu et al.,1999
IbeA	Huang et al.,2001
CNF1	Yao et al., 2006
aslA	Yao et al., 2006
fhuA	Yao et al., 2006
fyuA	Yao et al., 2006
hlyABCD	Yao et al., 2006
hlyE	Yao et al., 2006
iha	Yao et al., 2006
iroN	Yao et al., 2006
iucABCD	Yao et al., 2006
iutA	Yao et al., 2006
malX	Yao et al., 2006
ompA	Yao et al., 2006
papA	Yao et al., 2006
flu	Yao et al., 2006
traJ	Yao et al., 2006
sitABCD	Yao et al., 2006
apbE	Yao et al., 2006
blc	Yao et al., 2006
cutF	Yao et al., 2006
lgt	Yao et al., 2006
lnt	Yao et al., 2006
lolA	Yao et al., 2006
lpp	Yao et al., 2006
nlpB	Yao et al., 2006
nlpC	Yao et al., 2006
nlpD	Yao et al., 2006
nlpI	Yao et al., 2006
osmB	Yao et al., 2006
rlpAB	Yao et al., 2006
slyB	Yao et al., 2006
spr	Yao et al., 2006
vacJ	Yao et al., 2006
yafL	Yao et al., 2006
yajG	Yao et al., 2006
yehR	Yao et al., 2006
yfiO	Yao et al., 2006
yqhH	Yao et al., 2006
yhiU	Yao et al., 2006
bglH	Yao et al., 2006
btuB	Yao et al., 2006
cirA	Yao et al., 2006

fadL	Yao et al., 2006
fecA	Yao et al., 2006
fepA	Yao et al., 2006
nfrA	Yao et al., 2006
nmpC	Yao et al., 2006
ompC	Yao et al., 2006
ompF	Yao et al., 2006
ompG	Yao et al., 2006
ompN	Yao et al., 2006
ompT	Yao et al., 2006
ompW	Yao et al., 2006
pldA	Yao et al., 2006
sfmD	Yao et al., 2006
slp	Yao et al., 2006
yaeT	Yao et al., 2006
yaiV	Yao et al., 2006
ybiL	Yao et al., 2006
ycbS	Yao et al., 2006
yehB	Yao et al., 2006
yejO	Yao et al., 2006
yfiB	Yao et al., 2006
yiaD	Yao et al., 2006
yiaT	Yao et al., 2006
yjcP	Yao et al., 2006
yjhA	Yao et al., 2006
yjiK	Yao et al., 2006
yncD	Yao et al., 2006
yohG	Yao et al., 2006
ypjA	Yao et al., 2006
ytfM	Yao et al., 2006
